# An improved route to 19-substituted geldanamycins as novel Hsp90 inhibitors – potential therapeutics in cancer and neurodegeneration†
†Electronic supplementary information (ESI) available. See DOI: 10.1039/c3cc43457e



**DOI:** 10.1039/c3cc43457e

**Published:** 2013-06-17

**Authors:** Russell R. A. Kitson, Christopher J. Moody

**Affiliations:** a School of Chemistry , University of Nottingham , University Park , Nottingham , UK NG7 2RD . Email: c.j.moody@nottingham.ac.uk ; Fax: +44 (0)115 951 3564

## Abstract

19-Substituted geldanamycin derivatives are efficient Hsp90 inhibitors, without the toxicity associated with the other benzoquinone ansamycins, thus giving them potential for use as molecular therapeutics in cancer and neurodegeneration. Here a new method of synthesising these important compounds is reported, eliminating the need for toxic metals and metalloids.

## Abstract 2

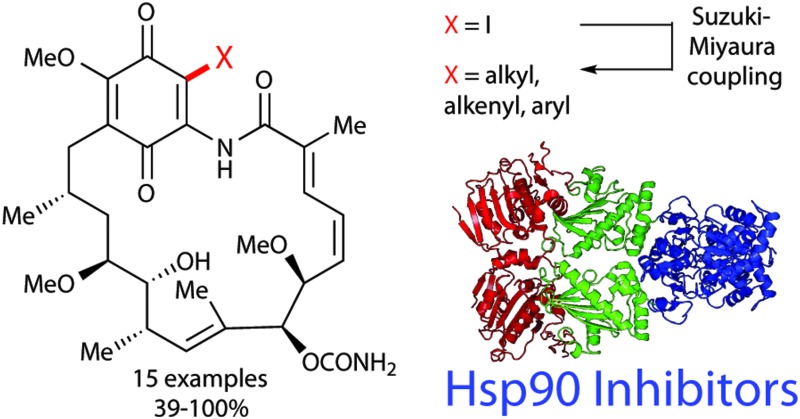
19-Substituted geldanamycin derivatives, now accessible by a ligand-free Suzuki–Miyaura protocol, are efficient Hsp90 inhibitors, without the toxicity associated with the other benzoquinone ansamycins, thus giving them potential for use as molecular therapeutics in cancer and neurodegeneration.

The benzoquinone ansamycin (BQA) polyketide geldanamycin **1** is arguably one of the most significant natural products to be isolated in the past fifty years. Initially found to be an efficient antibacterial agent,^1^ Neckers' 1994 discovery of geldanamycin's potent and specific inhibition of the molecular chaperone, heat shock protein 90 (Hsp90),^2^ prompted a veritable explosion of research in the area.^3^ Hsp90, one of the most abundant proteins in eukaryotic cells, has been shown to play a pivotal role in many oncogenic pathways,^4^ in addition to neurodegenerative diseases such as Alzheimer's and Parkinson's,^5^ and conditions such as HIV/AIDS^6^ and malaria.^7^ As a result, the enzyme has become one of the most attractive and widely studied molecular targets for small molecule inhibition, with over 15 inhibitors already in clinical trials as cancer therapeutics.^3,6,7^ Despite geldanamycin **1** providing an excellent lead for drug discovery, it was not progressed to the clinic, due to poor solubility and stability and, in particular, unacceptable liver toxicity. The more stable and soluble semi-synthetic geldanamycin derivatives 17-allylamino-17-demethoxygeldanamycin (17-AAG, Tanespimycin) **2**,^8^ and 17-*N*,*N*-dimethylethylenediamino-17-demethoxygeldanamycin (17-DMAG, Alvespimycin) **3** (ref. 8) (Fig. 1) were developed and advanced to clinical trials, although continuing difficulties with formulation and toxicity meant their progress was also halted.^9^

**Fig. 1:**
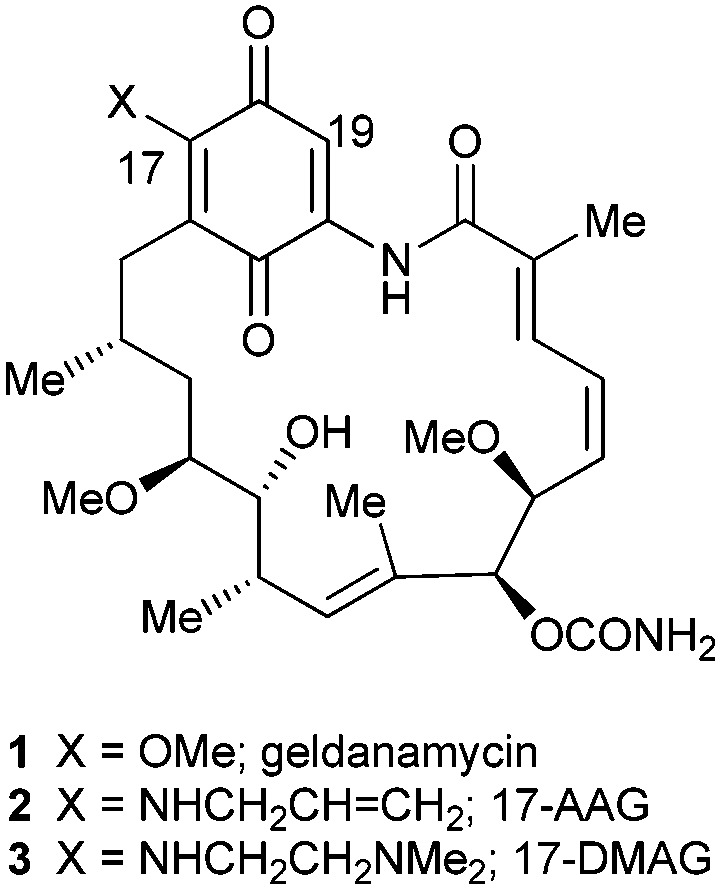
The Hsp90 inhibitors geldanamycin **1**, its aminoquinone analogues 17-allylamino-17-demethoxygeldanamycin (17-AAG) **2**, 17-*N*,*N*-dimethylethylenediamino-17-demethoxygeldanamycin (17-DMAG) **3**.

Following our previous work in the Hsp90 arena,^3,10^ we recently reported our efforts to address the toxicity associated with geldanamycin analogues.^11^ The introduction of a substituent at the 19-position of the quinone ring was found to completely suppress the conjugate addition of thiol nucleophiles, thought to be responsible for the liver toxicity,^12^ and render the 19-substituted BQAs (19-BQAs) **5** essentially non-toxic.^11^ Thus, the amino-quinone analogue 19-phenyl-17-AAG was found to have an IC_50_ greater than 20 μM against both normal human umbilical vein endothelial cells (HUVECs) and retinal pigmented epithelial cells (ARPE-19 cells).^11^ In addition it also caused a *trans* to *cis*-amide isomerisation,^13^ known to be required for binding to the Hsp90 protein to occur,^11^ whilst protein crystallography established that such 19-BQAs did indeed bind efficiently to the *N*-terminal ATP-binding site of Hsp90 (Fig. 2).^11^

**Fig. 2:**
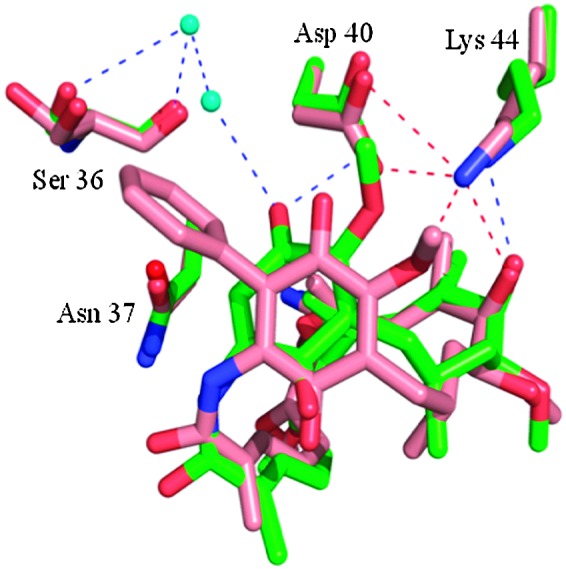
X-ray structure of 19-phenyl geldanamycin **5** bound in the ATP site of yeast Hsp90.^11^ Geldanamycin **1** (green) and 19-phenyl geldanamycin **5** (salmon) with Hsp90 (green and salmon residues, respectively). See PDB codes ; 1A4H (geldanamycin **1**) and ; 4ASF (19-phenyl geldanamycin **5**).

In order to access these 19-substituted geldanamycin derivatives, a palladium-catalysed Stille coupling was employed, utilising the readily accessible 19-iodogeldanamcyin **4**.^14^ This is a complex and challenging substrate, but suitable conditions were successfully developed, although in some cases the Stille couplings proved limited in scope, and were not scalable above 0.1 mmol without a reduction in yield. Additionally, the use of tin and the arsine ligands required for high yields, makes the coupling protocol relatively unattractive for the pharmaceutical industry. Herein we report our endeavours to develop a new method to access these biologically important 19-BQAs.

The palladium-catalysed Suzuki–Miyaura reaction^15^ is well known to be the coupling protocol of choice for industry in view of the large array of boronic acid and ester coupling partners commercially available, its high yields and its relatively benign starting materials and by-products. In view of this, we were attracted to the use of a Suzuki–Miyaura protocol to access 19-BQAs. Early efforts under standard sets of conditions were unsuccessful.^11^ However, a 2004 report of the coupling of 17-triflyloxy-BQAs with boronic acids,^18^ employing the Neel modification of the Suzuki–Miyaura protocol (‘ligand-free’ Pd_2_(dba)_3_·CHCl_3_ as the catalyst, CsBr for ligand-exchange with the palladium–triflate complex, CsF as the base and 1,4-dioxane as the solvent),^19^ gave some hope. Encouraged, we applied similar conditions to 19-iodogeldanamycin **4**, with the aim of accessing a range of 19-BQAs (Scheme 1).

**Scheme 1:**
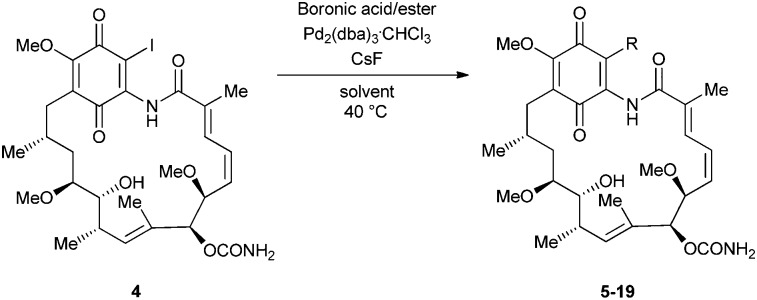
Scope of the Suzuki–Miyaura coupling reaction; synthesis of 19-substituted geldanamycins **5–19**.

Our initial attempts employed benzeneboronic acid as coupling partner, Pd_2_(dba)_3_·CHCl_3_, CsF and 1,4-dioxane (the caesium bromide was eliminated in view of our use of the iodide as opposed to the triflate) and we were delighted to observe that 19-phenylgeldanamycin **5** was obtained in an excellent 91% yield, a slight increase over our Stille protocol (85%).^11^ Following this initial success, we optimised the coupling procedure in terms of solvent and temperature and investigated the use of different phenyl-boron coupling partners (for optimisation studies, see the ESI†).

Following our optimisation studies, we investigated the scope of the methodology in terms of the boron coupling partner (Table 1). Despite our success with the phenyl group transfer, cross-coupling reactions to install a methyl group (entry 3) were less efficient, with a moderate yield of 39% achievable using methylboronic acid (the reaction with trimethylboroxine gave no product), although importantly, it eliminated the need to handle tetramethyl stannane. The new protocol also allowed access to 19-alkyl-BQAs previously unobtainable *via* the Stille method, exemplified by entry 4, for which an unoptimised 19% yield was achieved for a particularly troublesome isopropyl coupling, and entry 5, where an excellent yield of 19-allyl-geldanamycin **8** was obtained. Coupling of a vinyl group was achieved in good yield with both the pinacol and MIDA^17^ boronates (entry 6). However, reactions to couple more complex vinylic substituents gave yields in excess of 90% (entries 7 and 8). Additionally, dihydrofuryl and dihydropyranyl groups were successfully coupled in good yield, with the former being obtained as the hydrolysed form **12** (entries 9 and 10). Significantly, the new method was found to be greatly superior to the Stille protocol for the vast majority of reactions with aromatic coupling partners (entries 11–16). Those with electron-rich aromatic groups gave excellent yields, whilst electron deficient coupling partners also performed well, giving the 2-nitrophenyl- and 4-acetylphenyl-geldanamycin derivatives **17** and **18** in 64 and 65% yield, respectively. The work-up and purification for the new approach was found to be significantly easier than for the Stille protocol. Rather than requiring repeated washing (saturated aqueous LiCl solution) to remove the DMF, followed by chromatography using 10% potassium carbonate/silica gel^20^ (with subsequent treatment of all glassware for tin contamination), our new procedure simply required the concentration of the reaction mixture, followed by straightforward silica gel chromatography.

**Table 1:** Scope of the Suzuki–Miyaura coupling reaction;^*a*^ synthesis of 19-substituted geldanamycins **5–19**

Entry	R	Product	Yield/%	Stille yield^*f*^/%^11^
1	Ph	**5**	91	85
2^*b*^	Ph	**5**	Quant	85
3	Me	**6**	39 (29^*c*^)	86
4	i-Pr	**7**	19	0
5^*b*^	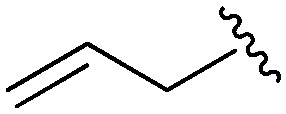	**8**	81	0
6^*b*^	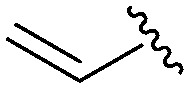	**9**	59 (54^*d*^)	76
7^*b*^	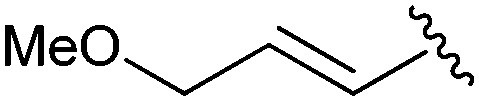	**10**	Quant	—
8^*b*^	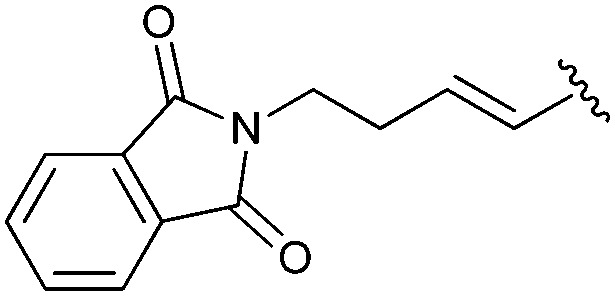	**11**	90	—
9^*b*^ ^,^ ^*e*^	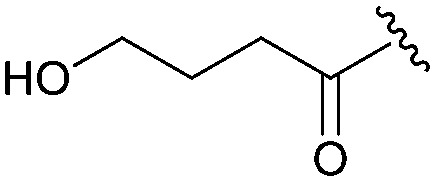	**12**	53	—
10^*b*^	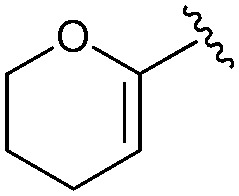	**13**	46	—
11	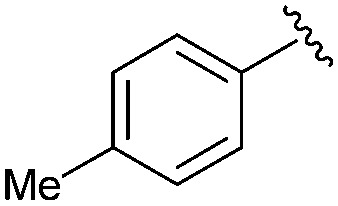	**14**	Quant	—
12	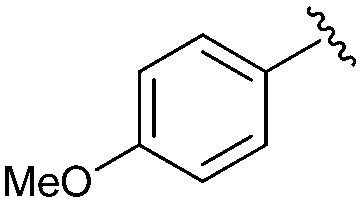	**15**	95	56
13	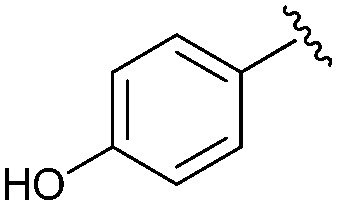	**16**	81	—
14	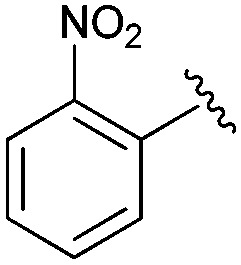	**17**	64	—
15	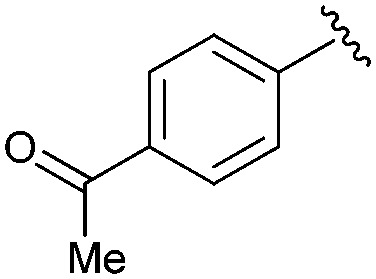	**18**	65	—
16	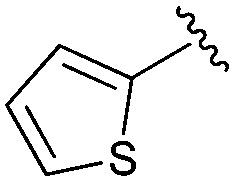	**19**	73	94

^*a*^Reactions performed at 0.02–0.04 M in 1,4-dioxane with 2.0 eq. boronic acid, 5 mol% Pd_2_(dba)_3_·CHCl_3_ and 2.0 eq. of CsF at 40 °C for 16 h.

^*b*^Performed with 2.0 eq. RB(pin) in 1,4-dioxane/H_2_O (9 : 1).

^*c*^Performed with 2.0 eq. MeBF_3_^–^K^+^ in i-PrOH/H_2_O (9 : 1) with 3.0 eq. of Et_3_N.^16^

^*d*^Performed with 2.0 eq. vinylboronic acid MIDA boronate.

^*e*^Performed with 2.0 eq. 2,3-dihydro-5-furylboronic acid pinacol ester.

^*f*^Stille reactions were performed using Me_4_Sn for methyl couplings and RSnBu_3_ for all other couplings under the conditions outlined in ref. 11 [dba = dibenzylideneacetone, B(pin) = 4,4,5,5-tetramethyl-1,3,2-dioxaborolane, MIDA = *N*-methyliminodiacetic acid].^17^

In summary, a new Suzuki–Miyaura based protocol has been developed for accessing important 19-substituted geldanamycin Hsp90 inhibitors, compounds which we have previously shown to be significantly less toxic to normal endothelial and epithelial cell systems than their parent quinones^11^ and, as such, have considerable potential as therapeutic agents. The novel BQAs obtained by this method are currently undergoing biological evaluation in both the therapy of cancer and neurodegenerative diseases. The new methodology is complementary to our previous Stille approach and, significantly, eliminates the need for the use and disposal of toxic metals or metalloids. These factors, in addition to the much wider commercial availability of boron coupling partners, make the new methodology much more attractive to the pharmaceutical industry and the wider chemical community, whilst making important bioactive compounds more accessible.

This work was supported by Parkinson's UK (R.R.A.K. and C.J.M.). The authors also thank S. Aslam (UoN, NMR), M. Cooper and G. Coxhill (UoN, MS) for technical assistance and A. Jolibois for a sample of (*E*)-2-(4-(4,4,5,5-tetramethyl-1,3,2-dioxaborolan-2-yl)but-3-en-1-yl)isoindoline-1,3-dione.

## References

[cit1] DeBoer C., Meulman P. A., Wnuk R. J., Peterson D. H. (1970). J. Antibiot..

[cit2] Whitesell L., Mimnaugh E. G., Decosta B., Myers C. E., Neckers L. M. (1994). Proc. Natl. Acad. Sci. U. S. A..

[cit3] Kitson R. R. A., Moody C. J. (2013). J. Org. Chem..

[cit4] McDonald E., Workman P., Jones K. (2006). Curr. Top. Med. Chem..

[cit5] Adachi H., Katsuno M., Waza M., Minamiyama M., Tanaka F., Sobue G., Aridon P., Geraci F., Turturici G., D'Amelio M., Savettieri G., Sconzo G., Gallo K. A., Kalia S. K., Kalia L. V., McLean P. J., Luo G.-R., Chen S., Le W.-D., Sajjad M. U., Samson B., Wyttenbach A. (2009). Int. J. Hyperthermia.

[cit6] Brenner B. G., Wainberg Z., Roesch F., Meziane O., Kula A., Nisole S., Porrot F., Anderson I., Mammano F., Fassati A., Marcello A., Benkirane M., Schwartz O., Vozzolo L., Loh B., Gane P. J., Tribak M., Zhou L. H., Anderson I., Nyakatura E., Jenner R. G., Selwood D., Fassati A. (2001). Expert Opin. Biol. Ther..

[cit7] Acharya P., Kumar R., Tatu U., Sharma Y. D., Wider D., Peli-Gulli M. P., Briand P. A., Tatu U., Picard D. (2007). Mol. Biochem. Parasitol..

[cit8] Schnur R. C., Corman M. L., Gallaschun R. J., Cooper B. A., Dee M. F., Doty J. L., Muzzi M. L., DiOrio C. I., Barbacci E. G., Schnur R. C., Corman M. L., Gallaschun R. J., Cooper B. A., Dee M. F., Doty J. L., Muzzi M. L., Moyer J. D., DiOrio C. I. (1995). J. Med. Chem..

[cit9] Banerji U., O'Donnell A., Scurr M., Pacey S., Stapleton S., Asad Y., Simmons L., Maloney A., Raynaud F., Campbell M., Walton M., Lakhani S., Kaye S., Workman P., Judson I., Goetz M. P., Toft D., Reid J., Ames M., Stensgard B., Safgren S., Adjei A. A., Sloan J., Atherton P., Vasile V., Salazaar S., Adjei A., Croghan G., Erlichman C., Ramanathan R. K., Trump D. L., Eiseman J. L., Belani C. P., Agarwala S. S., Zuhowski E. G., Lan J., Potter D. M., Ivy S. P., Ramalingam S., Brufsky A. M., Wong M. K. K., Tutchko S., Egorin M. J., Sausville E. A., Tomaszewski J. E., Ivy P. (2005). J. Clin. Oncol..

[cit10] Day J. E. H., Blake A. J., Moody C. J., Day J. E. H., Sharp S. Y., Rowlands M. G., Aherne W., Hayes A., Raynaud F. I., Lewis W., Roe S. M., Prodromou C., Pearl L. H., Workman P., Moody C. J., Day J. E. H., Sharp S. Y., Rowlands M. G., Aherne W., Lewis W., Roe S. M., Prodromou C., Pearl L. H., Workman P., Moody C. J., Day J. E. H., Sharp S. Y., Rowlands M. G., Aherne W., Workman P., Moody C. J., McErlean C. S. P., Proisy N., Davis C. J., Boland N. A., Sharp S. Y., Boxall K., Slawin A. M. Z., Workman P., Moody C. J., Proisy N., Sharp S. Y., Boxall K., Connelly S., Roe S. M., Prodromou C., Slawin A. M. Z., Pearl L. H., Workman P., Moody C. J. (2009). Synlett.

[cit11] Kitson R. R. A., Chuan C.-H., Xiong R., Williams H. E. L., Davis A. L., Lewis W., Dehn D. L., Siegel D., Roe S. M., Prodromou C., Ross D., Moody C. J. (2013). Nat. Chem..

[cit12] Cysyk R. L., Parker R. J., Barchi J. J., Steeg P. S., Hartman N. R., Strong J. A., Guo W., Reigan P., Siegel D., Ross D., Lang W. S., Caldwell G. W., Li J., Leo G. C., Jones W. J., Masucci J. A. (2006). Chem. Res. Toxicol..

[cit13] Jez J. M., Chen J. C. H., Rastelli G., Stroud R. M., Santi D. V., Lee Y. S., Marcu M. G., Neckers L., Onuoha S. C., Mukund S. R., Coulstock E. T., Serengovà B., Shaw J., McLaughlin S. H., Jackson S. E., Reigan P., Siegel D., Guo W. C., Ross D., Thepchatri P., Eliseo T., Cicero D. O., Myles D., Snyder J. P. (2003). Chem. Biol..

[cit14] Sasaki K. and Inoue Y., German Patent No. Ger. Offen., 3006097, 1980.

[cit15] Miyaura N., Yamada K., Suzuki A. (1979). Tetrahedron Lett..

[cit16] Molander G. A., Katona B. W., Machrouhi F. (2002). J. Org. Chem..

[cit17] Knapp D. M., Gillis E. P., Burke M. D. (2009). J. Am. Chem. Soc..

[cit18] Le Brazidec J.-Y., Kamal A., Busch D., Thao L., Zhang L., Timony G., Grecko R., Trent K., Lough R., Salazar T., Khan S., Burrows F., Boehm M. F. (2004). J. Med. Chem..

[cit19] Neel D. A., Jirousek M. R., McDonald J. H. (1998). Bioorg. Med. Chem. Lett..

[cit20] Harrowven D. C., Curran D. P., Kostiuk S. L., Wallis-Guy I. L., Whiting S., Stenning K. J., Tang B., Packard E., Nanson L. (2010). Chem. Commun..

