# Supplementary material for: An improved route to 19-substituted geldanamycins as novel Hsp90 inhibitors – potential therapeutics in cancer and neurodegeneration
Source: Chem Commun (Camb). 2013 Jun 17;49(76):8441–3. doi: 10.1039/c3cc43457e (PMC3835074; doi:10.1039/c3cc43457e)
Supplement: Supplementary file 1 [file cc-049-c3cc43457e-s001.pdf]

This journal is © The Royal Society of Chemistry 2013

Supplementary Information

An Improved Route to 19-Substituted Geldanamycins as Novel Hsp90 Inhibitors - Potential Therapeutics in Cancer and Neurodegeneration

Russell R. A. Kitson and Christopher J. Moody*

School of Chemistry, University of Nottingham, University Park, Nottingham NG7 2RD, U.K.

Fax: (+44) 115 951 3564; E-mail: c.j.moody@nottingham.ac.uk

This journal is © The Royal Society of Chemistry 2013

Table of Contents

	Page
**Experimental Details**	S3
Suzuki-Miyaura Coupling Optimisation	S3
General Experimental Details	S5
Prepared Compounds	S7
General Procedure 1: Preparation of 19-substituted-geldanamycin derivatives.	S7
References	S28
**NMR Spectra**	S29

Experimental Details

Suzuki-Miyaura Coupling Optimisation

The coupling proceeded efficiently in dioxane/water, albeit with a decrease in the overall yield from the corresponding anhydrous reaction (Table 1, Entry 2). Indeed 1,4-dioxane was found to be the reaction medium of choice, with significantly diminished yields obtained with the vast majority of other solvents tested (Entries 3-6, 9 and 10). The exception in this case was alcoholic solvents, for which excellent yields of 84% and 87% were obtained for methanol and isopropanol, respectively (Entries 7 and 8). We also attempted the coupling of a phenyl group, utilising the alternative boronate species outlined in Entries 11-14. Reactions using the more stable phenyl MIDA boronate[1] and potassium phenyltrifluoroborate[2] were found to be moderately successful. However, we were delighted to find that performing the reactions with the corresponding pinacol ester gave 19-phenylgeldanamycin **8** in quantitative yield after simple chromatography (Entry 11).

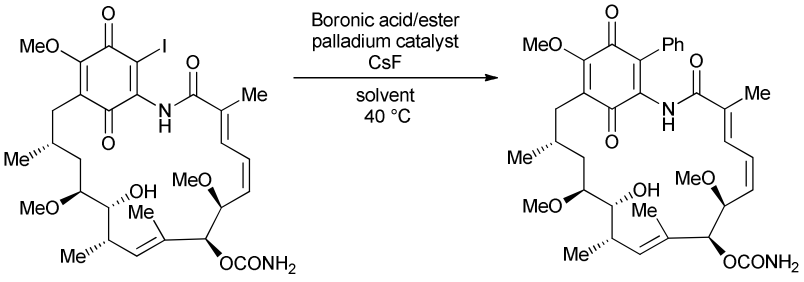

4 **5**

Scheme 1. Optimisation of the Suzuki-Miyuara coupling approach to 19-substituted BQAs.

Table 1. Optimisation of the Suzuki-Miyaura coupling reaction.[a]

Entry	Solvent	Boron species	**8** Yield/%	Recovered **1**/%
1[b]	1,4-dioxane	$PhB(OH)_2$	91	<5
2[b]	1,4-dioxane/H_2O (9:1)	$PhB(OH)_2$	78	14

This journal is © The Royal Society of Chemistry 2013

3	THF	$PhB(OH)_2$	26	54
4	THF/H_2O (9:1)	$PhB(OH)_2$	57	29
5[c]	CH_2Cl_2	$PhB(OH)_2$	23	31
6	MeCN	$PhB(OH)_2$	12	<5
7	MeOH	$PhB(OH)_2$	84	<5
8	*i*-PrOH	$PhB(OH)_2$	87	<5
9	*i*-PrOH/H_2O (9:1)	$PhB(OH)_2$	56	27
10	DMF	$PhB(OH)_2$	4	<5
11	1,4-dioxane/H_2O (9:1)	PhB(pin)	Quant.	<5
12	1,4-dioxane/H_2O (9:1)	PhB(MIDA)	20	8
13[d]	*i*-PrOH/H_2O (9:1)	$PhBF_3^-K^+$	68	<5
14[d,e]	*i*-PrOH/H_2O (9:1)	$PhBF_3^-K^+$	27	71

[a] All reactions were conducted at a concentration of 0.02-0.04 M in the solvent specified with 2.0 eq. of the boron coupling partner, 5 mol% $Pd_2(dba)_3 \cdot CHCl_3$ and 2.0 eq. of caesium fluoride at 40 ºC for 16 h. [b] The reaction was heated to 40 ºC for 4 h. [c] The reaction was heated to reflux for 16 h. [d] The reaction was performed with 3.0 eq. of triethylamine.[2] [b] The reaction was performed with 5 mol% $PdCl_2(dppf) \cdot CH_2Cl_2$. dba = Dibenzylideneacetone, B(pin) = 4,4,5,5-tetramethyl-1,3,2-dioxaborolane, MIDA = *N*-methyliminodiacetic acid,[1] dppf = 1,1'-bis(diphenylphosphino)ferrocene.

This journal is © The Royal Society of Chemistry 2013

General Experimental Details.

Except where specified, all reagents were purchased from commercial sources and were used without further purification. 1,4-Dioxane was distilled from sodium-benzophenone ketyl immediately before use. (*E*)-2-(4-(4,4,5,5-tetramethyl-1,3,2-dioxaborolan-2-yl)but-3-en-1-yl)isoindoline-1,3-dione was prepared *via* a Mitsunobu-Wang hydroboration procedure using homo-propargyl alcohol, phthalimide and pinacol borane (see references 3 and 4 for details).

Thin layer chromatography (TLC) was performed using Merck Kieselgel $60GF_{254}$ pre-coated aluminium-backed plates. The compounds were visualised by UV light (254 nm) and basic aqueous potassium permanganate. Flash chromatography was performed at medium pressure using slurry packed Davisil silica gel 35-70 µm, 60 Å with the eluant specified. Light petroleum is the fraction with bp 40-60 °C.

Infrared spectra were recorded on a Perkin Elmer 1600 series FT-IR spectrometer using NaCl solution cells in chloroform. ^{1}H NMR and ^{13}C NMR spectra were recorded on a Bruker Avance III-400, Bruker Avance 400 or Bruker DPX 400 spectrometer, operating at 400 MHz and 100 MHz, respectively, or a Bruker Avance III-500 spectrometer, operating at 500 MHz and 125 MHz, respectively. All spectroscopic data were acquired at 295 K. Chemical shifts are quoted in parts per million (ppm), using the residual solvent peak as an internal standard (2.50 ppm [^{1}H NMR] for DMSO-H_6 and 39.52 ppm [^{13}C NMR] for DMSO-d_6). Coupling constants (*J*) are reported in Hz. Multiplicity abbreviations used: s singlet, d doublet, t triplet, q quartet, m multiplet. Signal assignment was accomplished by analysis of DEPT, COSY, NOESY, HMBC and HSQC experiments where necessary.

This journal is © The Royal Society of Chemistry 2013

Low and high-resolution mass spectra were obtained for all novel compounds. Electrospray ionization (ESI) and high resolution mass spectrometric (HRMS) analyses were measured on a Bruker MicroTOF spectrometer.

Specific rotation values were measured on an ADP-440 digital polarimeter using a sodium lamp at 589 nm. Melting points were determined using a Riechert-Kofler hot stage apparatus and are uncorrected.

The numbering and naming of compounds does not conform to IUPAC rules, instead conforming to the traditional numbering system for the ansamycins.

Prepared Compounds

(4E,6Z,8S,9S,10E,12S,13R,14S,16R)-13-Hydroxy-8,14,19-trimethoxy-,10,12,16,21-pentamethyl-3,20,22-trioxo-2-azabicyclo[16.3.1]docosa-1(21),4,6,10,18-pentaen-9-yl carbamate

***[19-Phenylgeldanamycin]* 5**

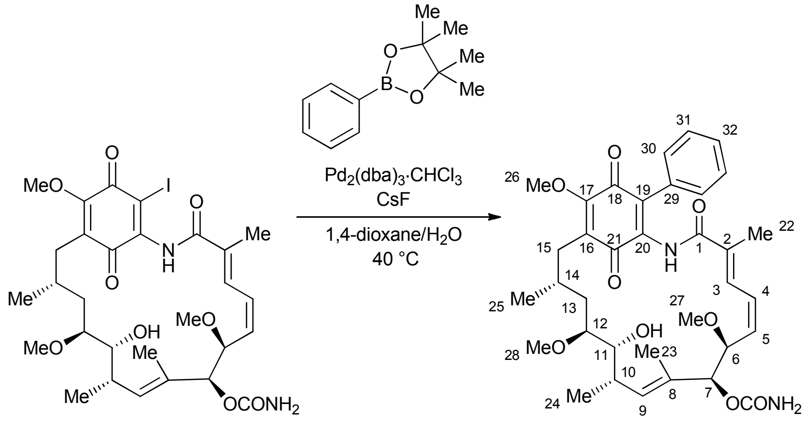

General Procedure 1- Preparation of 19-substituted-geldanamycin derivatives:

A stirred solution of 19-iodogeldanamycin **4**[5] (19 mg, 0.028 mmol, 1.0 eq.), phenylboronic acid pinacol ester (11 mg, 0.054 mmol, 2.0 eq.), *tris*-(dibenzylideneacetone)dipalladium(0) chloroform complex (1.4 mg, 0.001 mmol, 5 mol%) and caesium fluoride (8 mg, 0.054 mmol, 2.0 eq.) in 1,4-dioxane/water (9:1, 2 mL [ca. 0.02-0.04 M]) was sparged with argon for 20 min. The reaction mixture was heated to 40 °C for 16 h, before being concentrated *in vacuo*. The residue was purified by flash chromatography on silica gel, eluting with ethyl acetate to give the *title compound* **5** (18 mg, quantitative yield) as an orange solid; TLC R_f = 0.37 (ethyl acetate, det: $KMnO_4$/Δ); mp 232-233 °C; $[a]_D^{24}$ +190.4 (*c* 0.12, $CHCl_3$); δ_H (500 MHz; DMSO-d_6) 9.51 (1H, s, NH), 7.52 (2H, ddt, *J* 8.4, 7.2, 1.4, H-31), 7.45 (1H, tt, *J* 7.2, 1.4, H-32), 7.38

(2H, dd, *J* 8.4, 1.4, H-30), 6.54 (1H, d, *J* 11.6, H-3), 6.41 (1H, t, *J* 11.6, H-4), 6.46-6.21 (2H, br. s, NH_2), 5.31 (1H, dd, *J* 11.6, 10.7, H-5), 5.18 (1H, d, *J* 10.4, H-9), 4.90 (1H, d, *J* 9.0, H-7), 4.37 (1H, d, *J* 4.3, OH), 4.00 (1H, dd, *J* 10.7, 9.0, H-6), 3.97 (3H, s, OMe-26), 3.47 (1H, ddd, *J* 9.3, 4.3, 2.9, H-11), 3.20 (3H, s, OMe-28), 3.11 (3H, s, OMe-27), 2.82 (1H, dt, *J* 9.4, 2.9, H-12), 2.54 (1H, dd, *J* 12.3, 5.9, H-15), 2.41 (1H, dd, *J* 12.3, 4.4, H-15), 2.17-2.03 (2H, m, H-10+14), 1.86 (3H, s, Me-22), 1.45 (1H, ddd, *J* 13.7, 9.4, 4.3, H-13), 1.24 (3H, s, Me-23), 0.87 (3H, d, *J* 6.5, Me-24), 0.65 (1H, ddd, *J* 13.7, 11.4, 2.9, H-13), 0.65 (3H, d, *J* 6.7, Me-25). Data are consistent with those reported in the literature.[6]

(4E,6Z,8S,9S,10E,12S,13R,14S,16R)-13-Hydroxy-8,14,19-trimethoxy-,10,12,16,21-pentamethyl-3,20,22-trioxo-2-azabicyclo[16.3.1]docosa-1(21),4,6,10,18-pentaen-9-yl carbamate

***[19-Methylgeldanamycin]* 6**

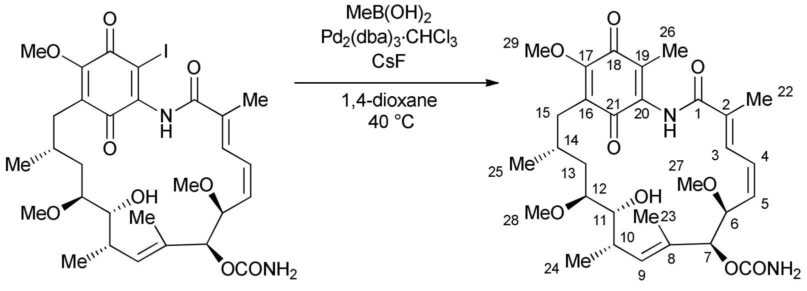

The reaction was carried out according to general procedure 1, using 19-iodogeldanamycin **4**[5] (49 mg, 0.071 mmol, 1.0 eq.), *tris*-(dibenzylideneacetone)dipalladium(0) chloroform complex (4 mg, 0.004 mmol, 5 mol%) and caesium fluoride (22 mg, 0.143 mmol, 2.0 eq.), differing only in those methylboronic acid (9 mg, 0.143 mmol, 2.0 eq.) was used instead of phenyl boronic acid pinacol ester and 1,4-dioxane (2 mL) was used instead of 1,4-dioxane/water.

Purification by flash chromatography on silica gel, eluting with 1:2 light petroleum/ethyl acetate → ethyl acetate gave the *title compound* **6** (16 mg, 39%) as a yellow solid; TLC R_f = 0.29 (1:2 light petroleum/ethyl acetate, det: $KMnO_4$/Δ); mp 138-141 °C; $[a]_D^{22}$ +80.8 (*c* 0.14, $CHCl_3$); δ_H (400 MHz; DMSO-d_6) 9.58 (1H, s, NH), 6.45-6.23 (2H, br. s, NH_2), 6.33 (1H, dd, *J* 12.1, 10.5, H-4), 6.27 (1H, d, *J* 12.1, H-3), 5.21 (1H, t, *J* 10.5, H-5), 5.14 (1H, d, *J* 9.9, H-9), 4.86 (1H, d, *J* 9.3, H-7), 4.37 (1H, d, *J* 4.1, OH), 3.96 (1H, dd, *J* 10.5, 9.3, H-6), 3.94 (3H, s, OMe-29), 3.46 (1H, ddd, *J* 9.1, 4.1, 2.5, H-11), 3.18 (3H, s, OMe-28), 3.04 (3H, s, OMe-27), 2.77 (1H, dt, *J* 8.7, 2.5, H-12), 2.45 (1H, dd, *J* 12.1, 5.2, H-15), 2.35 (1H, dd, *J* 12.1, 3.6, H-15), 2.11-2.04 (2H, m, H-10+14), 2.01 (3H, s, Me-26), 1.83 (3H, s, Me-22), 1.40 (1H, ddd, *J* 13.9, 8.7, 4.1, H-13), 1.18 (3H, s, Me-23), 0.87 (3H, d, *J* 6.3, Me-24), 0.65 (1H, ddd, *J* 13.9, 11.4, 2.5, H-13), 0.59 (3H, d, *J* 6.7, Me-25). Data are consistent with those reported in the literature.[6]

(4E,6Z,8S,9S,10E,12S,13R,14S,16R)-13-Hydroxy-21-isopropyl-8,14,19-trimethoxy-4,10,12,16-tetramethyl-3,20,22-trioxo-2-azabicyclo[16.3.1]docosa-1(21),4,6,10,18-pentaen-9-yl carbamate

***[19-isoPropylgeldanamycin]* 7**

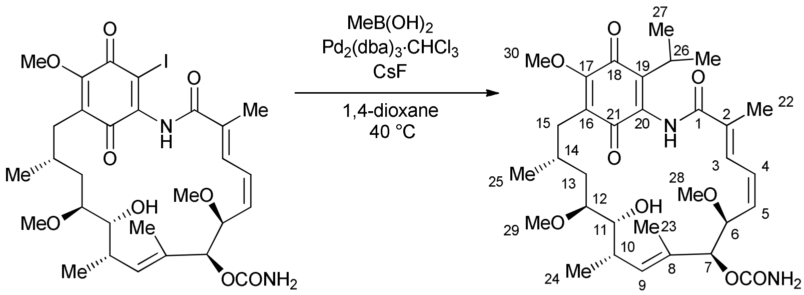

4 **7**

This journal is © The Royal Society of Chemistry 2013

The reaction was carried out according to general procedure 1, using 19-iodogeldanamycin **4**[5] (55 mg, 0.080 mmol, 1.0 eq.), *tris*-(dibenzylideneacetone)dipalladium(0) chloroform complex (4 mg, 0.004 mmol, 5 mol%) and caesium fluoride (24 mg, 0.160 mmol, 2.0 eq.), differing only in that isopropylboronic acid (14 mg, 0.160 mmol, 2.0 eq.) was used instead of phenyl boronic acid pinacol ester and 1,4-dioxane (2 mL) was used instead of 1,4-dioxane/water. Purification by flash chromatography on silica gel, eluting with 1:2 light petroleum/ethyl acetate → ethyl acetate gave the *title compound* **7** (9 mg, 19%) as a yellow solid; TLC R_f = 0.15 (1:2 light petroleum/ethyl acetate, det: $KMnO_4$/Δ); mp 114-115 °C; $[a]_D^{22}$ −90.0 (*c* 0.03, $CHCl_3$); (Found: M+Na$^+$, 625.3062. $C_{32}H_{46}N_2O_9$+Na$^+$, requires 609.3096); ν_{max} ($CHCl_3$)/cm^{-1} 3647, 3431, 2966, 2933, 1731, 1655, 1369, 1239; δ_H (500 MHz; DMSO-*d*$_6$) 9.70 (1H, s, NH), 6.63-6.13 (2H, br. s, NH_2), 6.36 (1H, dd, *J* 11.7, 10.6, H-4), 6.27 (1H, d, *J* 11.7, H-3), 5.24 (1H, dd, *J* 10.6, 9.9, H-5), 5.17 (1H, d, *J* 10.4, H-9), 4.88 (1H, d, *J* 9.2, H-7), 4.35 (1H, br. s, OH), 3.92 (3H, s, OMe-30), 3.89 (1H, dd, *J* 9.9, 9.2, H-6), 3.47 (1H, d, *J* 9.3, H-11), 3.25-3.17 (1H, m, H-26), 3.18 (3H, s, H-29), 3.02 (3H, s, H-28), 2.80-2.76 (1H, m, H-12), 2.46 (1H, dd, *J* 12.4, 5.7, H-15), 2.32 (1H, dd, *J* 12.4, 4.3, H-15), 2.13-2.00 (2H, m, H-10+14), 1.84 (3H, s, Me-22), 1.42 (1H, ddd, *J* 14.1, 9.8, 4.2, H-13), 1.31 (3H, d, *J* 6.0, H-27a), 1.29 (3H, d, *J* 6.7, H-27b), 1.26 (3H, s, Me-23), 1.25-1.22 (1H, m, H-13), 0.88 (3H, d, *J* 6.4, H-24), 0.67-0.58 (1H, m, H-13), 0.57 (3H, d, *J* 6.7, H-25); δ_C (125 MHz; DMSO-*d*$_6$) 184.6 (C=O-21), 182.9 (C=O-18), 173.7 (C=O-1), 157.4 (C-17), 156.3 (OC=ONH_2), 139.9 (C-20), 139.0 (C-2), 136.0 (C-19), 134.6 (CH-9), 130.5 (CH-5), 128.9 (C-8), 128.9 (CH-4), 128.7 (C-16), 123.2 (CH-3), 80.4 (CH-7), 80.0 (CH-12), 75.0 (CH-6), 71.8 (CH-11), 61.4 (Me-30), 56.1 (Me-29), 56.1 (Me-28), 35.3 (CH-10), 31.1 (CH_2-13), 29.9 (CH_2-15), 29.0 (CH-14), 27.5 (CH-26), 21.3 (Me-27a), 20.4 (Me-27b), 19.1 (Me-24), 19.1 (Me-25), 14.5 (Me-22), 12.4 (Me-23); *m/z* (ESI) 625 ([M+Na]$^+$, 100%).

(4E,6Z,8S,9S,10E,12S,13R,14S,16R)-21-Allyl-13-hydroxy-8,14,19-trimethoxy-4,10,12,16-tetramethyl-3,20,22-trioxo-2-azabicyclo[16.3.1]docosa-1(21),4,6,10,18-pentaen-9-yl carbamate

***[19-Allylgeldanamycin]* 8**

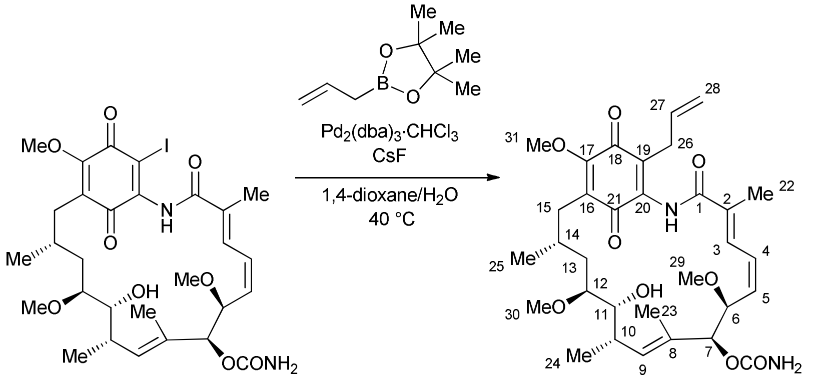

4 8

The reaction was carried out according to general procedure 1, using 19-iodogeldanamycin **4**[5] (34 mg, 0.050 mmol, 1.0 eq.), *tris*-(dibenzylideneacetone)dipalladium(0) chloroform complex (3 mg, 0.003 mmol, 5 mol%) and caesium fluoride (15 mg, 0.100 mmol, 2.0 eq.) in 1,4-dioxane/water (9:1, 2 mL), differing only in that allylboronic acid pinacol ester (17 mg, 0.100 mmol, 2.0 eq.) was used instead of phenyl boronic acid pinacol ester. Purification by flash chromatography on silica gel, eluting with 1:2 light petroleum/ethyl acetate → ethyl acetate gave the *title compound* **8** (24 mg, 81%) as a yellow solid; TLC R_f = 0.46 (ethyl acetate, det: $KMnO_4$/Δ); mp 105-106 °C; $[a]_D^{22}$ −36.2 (*c* 0.43, $CHCl_3$); (Found: M+Na$^+$, 601.3133. $C_{32}H_{45}N_2O_9$+H$^+$, requires 601.3120); ν_{max} ($CHCl_3$)/cm^{-1} 3547, 3432, 3006, 2935, 1729, 1682, 1602, 1370, 1240, 1104, 1054; δ_H (500 MHz; DMSO-*d*$_6$) 9.78 (1H, br. s, NH), (1H, d, *J* 12.2, H-3), 6.70-6.23 (2H, br. s, NH_2), 6.53 (1H, t, *J* 12.2, H-4), 5.58-5.47 (2H, m, H-5+27), 5.30 (1H, d, *J* 11.3, H-9), 5.21-5.12 (2H, m, H-28), 4.78 (1H, d, *J* 9.8, H-7), 4.50 (1H, d, *J* 3.7, OH), 4.18 (1H, t, *J* 9.8, H-6), 3.91 (3H, s, OMe-31), 3.40 (3H, s, H-30), 3.21 (3H, s, H-29),

3.16-3.13 (1H, m, H-12), 2.86 (1H, dd, *J* 13.8, 7.5, H-26), 2.69 (1H, dd, *J* 13.8, 7.1, H-26), 2.68-2.66 (1H, m, H-11), 2.58-2.54 (2H, m, H-15), 2.39-2.28 (1H, m, H-10), 2.07-1.93 (1H, m, H-14), 1.90 (3H, s, Me-22), 1.47 (3H, s, Me-23), 1.35-1.16 (1H, m, H-13), 0.94 (3H, d, *J* 6.7, H-24), 0.78 (3H, d, *J* 7.0, H-25), 0.30 (1H, t, *J* 12.7, H-13); δ_C (125 MHz; DMSO-d_6) 189.4 (C=O-21), 185.8 (C=O-18), 165.1 (C=O-1), 158.0 (C-17), 155.9 (OC=ONH$_2$), 137.2 (CH-9), 136.8 (CH-5), 133.7 (C-16), 132.1 (C-20), 131.8 (CH-27), 130.6 (CH-3), 129.1 (CH-19), 127.8 (CH-4), 126.0 (C-8), 123.7 (C-2), 120.4 (CH$_2$-28), 83.0 (CH-12), 78.9 (CH-7), 77.6 (CH-11), 76.4 (CH-6), 60.3 (Me-30), 59.4 (Me-31), 56.6 (Me-29), 36.9 (CH$_2$-13), 36.8 (CH$_2$-26), 32.1 (CH-10), 29.5 (CH$_2$-15), 28.1 (CH-14), 18.0 (Me-25), 13.8 (Me-24), 13.0 (Me-22), 11.9 (Me-23); *m/z* (ESI) 623 ([M+Na]$^+$, 100%).

(4E,6Z,8S,9S,10E,12S,13R,14S,16R)-13-Hydroxy-8,14,19-trimethoxy-4,10,12,16-tetramethyl-3,20,22-trioxo-21-vinyl-2-azabicyclo[16.3.1]docosa-1(21),4,6,10,18-pentaen-9-yl carbamate

***[19-Vinylgeldanamycin]* 9**

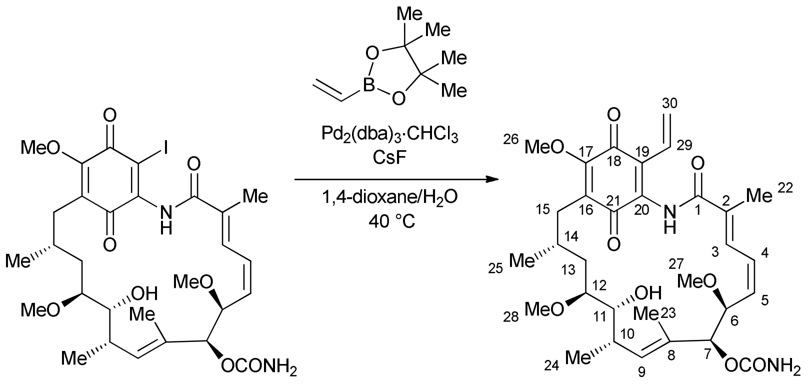

4 **9**

The reaction was carried out according to general procedure 1, using 19-iodogeldanamycin **4**[5] (20 mg, 0.029 mmol, 1.0 eq.), *tris*-(dibenzylideneacetone)dipalladium(0) chloroform complex

This journal is © The Royal Society of Chemistry 2013

(2 mg, 0.002 mmol, 5 mol%) and caesium fluoride (9 mg, 0.058 mmol, 2.0 eq.) in 1,4-dioxane/water (9:1, 2 mL), differing only in that vinylboronic acid pinacol ester (9 mg, 0.058 mmol, 2.0 eq.) was used instead of phenyl boronic acid pinacol ester. Purification by flash chromatography on silica gel, eluting with 1:2 light petroleum/ethyl acetate → ethyl acetate gave the *title compound* **9** (10 mg, 59%) as an orange solid; TLC R_f = 0.34 (ethyl acetate, det: $KMnO_4$/Δ); mp 172-173 °C; $[a]_D^{22}$ +21.9 (*c* 0.04, $CHCl_3$); δ_H (500 MHz; DMSO-d_6) 9.82 (1H, s, NH), 6.72 (1H, dd, *J* 17.7, 12.0, H-29), 6.60-6.17 (2H, br. s, NH_2), 6.35 (1H, dd, *J* 11.6, 9.2, H-4), 6.28 (1H, dd, *J* 17.7, 1.9, H-30), 6.27 (1H, d, *J* 9.2, H-3), 5.75 (1H, dd, *J* 12.0, 1.9, H-30), 5.23 (1H, dd, *J* 11.6, 10.0, H-5), 5.15 (1H, d, *J* 10.5, H-9), 4.86 (1H, d, *J* 10.0, H-7), 4.39 (1H, br. s, OH), 3.95 (3H, s, OMe-26), 3.88 (1H, t, *J* 10.0, H-6), 3.46 (1H, dd, *J* 9.2, 2.9, H-11), 3.18 (3H, s, H-28), 3.02 (3H, s, H-27), 2.78 (1H, dt, *J* 9.7, 2.9, H-12), 2.46 (1H, dd, *J* 12.5, 6.0, H-15), 2.35 (1H, dd, *J* 12.5, 4.4, H-15), 2.10-2.04 (2H, m, H-10+14), 1.84 (3H, s, Me-22), 1.40 (1H, ddd, *J* 14.0, 9.7, 3.4, H-13), 1.20 (3H, s, Me-23), 0.87 (3H, d, *J* 6.5, H-24), 0.65 (1H, ddd, *J* 14.0, 11.7, 2.9, H-13), 0.59 (3H, d, *J* 6.8, H-25). Data are consistent with those reported in the literature.[6]

(4E,6Z,8S,9S,10E,12S,13R,14S,16R)-13-Hydroxy-8,14,19-trimethoxy-21-((E)-3-methoxyprop-1-en-1-yl)-4,10,12,16-tetramethyl-3,20,22-trioxo-2-azabicyclo[16.3.1]docosa-1(21),4,6,10,18-pentaen-9-yl carbamate

***[19-(Methoxyprop-1-en-1-yl)geldanamycin]* 10**

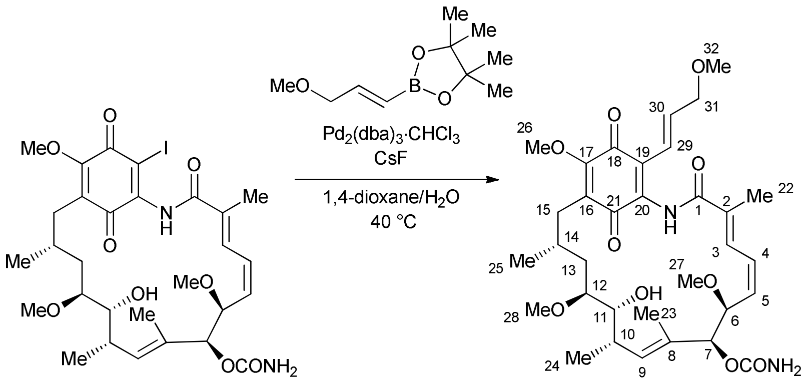

The reaction was carried out according to general procedure 1, using 19-iodogeldanamycin **4**[5] (36 mg, 0.052 mmol, 1.0 eq.), *tris*-(dibenzylideneacetone)dipalladium(0) chloroform complex (3 mg, 0.003 mmol, 5 mol%) and caesium fluoride (16 mg, 0.105 mmol, 2.0 eq.) in 1,4-dioxane/water (9:1, 2 mL), differing only in that *E*-3-methoxy-1-propenylboronic acid pinacol ester (21 mg, 0.105 mmol, 2.0 eq.) was used instead of phenyl boronic acid pinacol ester. Purification by flash chromatography on silica gel, eluting with 1:2 light petroleum/ethyl acetate → ethyl acetate gave the *title compound* **10** (33 mg, quantitative yield) as an orange solid; TLC R_f = 0.29 (ethyl acetate, det: $KMnO_4$/Δ); mp 166-167 °C; $[a]_D^{22}$ +257.0 (*c* 0.64, $CHCl_3$); (Found: M+Na$^+$, 653.3054. $C_{33}H_{46}N_2O_{10}$+Na$^+$, requires 653.3045); ν_{max} ($CHCl_3$)/cm^{-1} 3546, 3431, 3002, 1731, 1664, 1582, 1368, 1254, 1054; δ_H (500 MHz; DMSO-d_6) 9.79 (1H, s, NH), 6.85 (1H, dt, *J* 16.2, 5.5, H-30), 6.61 (1H, dt, *J* 16.2, 1.7, H-29), 6.47-6.19 (2H, br. s, NH_2), 6.36 (1H, dd, *J* 11.9, 10.6, H-4), 6.26 (1H, d, *J* 11.9, H-3), 5.24 (1H, dd, *J* 10.6, 10.0, H-5), 5.16 (1H, d, *J* 10.0, H-9), 4.87 (1H, d, *J* 9.3, H-7), 4.40 (1H, d, *J* 4.2, OH), 4.14 (2H, dd, *J* 5.5, 1.7, H-31), 3.95 (3H, s, OMe-26), 3.87 (1H, dd, *J* 10.0, 9.3, H-6), 3.47 (1H, ddd, *J* 9.7, 4.2, 2.7, H-11), 3.36 (3H, s, H-32), 3.19 (3H, s, H-28), 3.02 (3H, s, H-27), 2.79 (1H, dt, *J* 9.1, 2.7, H-12), 2.47 (1H, dd, *J* 12.6, 6.0, H-15), 2.36 (1H, dd, *J* 12.6, 3.9, H-15), 2.13-2.04 (2H, m, H-10+14), 1.85 (3H, s, Me-22), 1.42 (1H, ddd, *J* 14.0, 9.1, 4.2, H-13), 1.20 (3H, s, Me-23), 0.88 (3H, d, *J* 6.5, H-24), 0.67 (1H, td, *J* 14.0, 2.7, H-13), 0.60 (3H, d, *J*

6.8, H-25); δ_C (125 MHz; DMSO-d_6) 183.7 (C=O-21), 181.8 (C=O-18), 173.4 (C=O-1), 156.5 (C-17), 155.7 (OC=ONH$_2$), 138.5 (C-20), 138.0 (C-2), 137.7 (CH-30), 134.3 (CH-9), 130.2 (CH-5), 128.7 (C-16), 128.3 (C-8), 128.2 (CH-4), 124.5 (C-19), 122.8 (CH-3), 119.7 (CH-29), 79.9 (CH-7), 79.5 (CH-12), 74.3 (CH-6), 72.5 (CH$_2$-31), 71.5 (CH-11), 60.8 (Me-26), 57.6 (Me-32), 55.6 (Me-28), 55.5 (Me-27), 34.8 (CH-10), 30.5 (CH$_2$-13), 29.4 (CH$_2$-15), 28.4 (CH-14), 18.7 (Me-25), 18.5 (Me-24), 13.8 (Me-22), 11.3 (Me-23); *m/z* (ESI) 653 ([M+Na]$^+$, 100%).

***(4E,6Z,8S,9S,10E,12S,13R,14S,16R)-21-((E)-4-(1,3-Dioxoisoindolin-2-yl)but-1-en-1-yl)-13-hydroxy-8,14,19-trimethoxy-4,10,12,16-tetramethyl-3,20,22-trioxo-2-azabicyclo[16.3.1]docosa-1(21),4,6,10,18-pentaen-9-yl carbamate [19-((E)-4-(1,3-Dioxoisoindolin-2-yl)but-1-en-1-yl))geldanamycin]* 11**

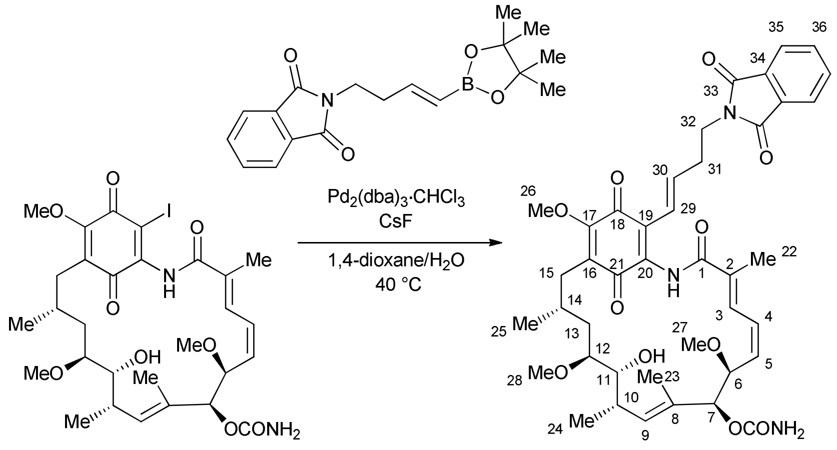

4 11

The reaction was carried out according to general procedure 1, using 19-iodogeldanamycin **4**[5] (28 mg, 0.041 mmol, 1.0 eq.), *tris*-(dibenzylideneacetone)dipalladium(0) chloroform complex (2 mg, 0.002 mmol, 5 mol%) and caesium fluoride (13 mg, 0.083 mmol, 2.0 eq.) in 1,4-dioxane/water (9:1, 2 mL), differing only in that (*E*)-2-(4-(4,4,5,5-tetramethyl-1,3,2-

This journal is © The Royal Society of Chemistry 2013

dioxaborolan-2-yl)but-3-en-1-yl)isoindoline-1,3-dione (27 mg, 0.083 mmol, 2.0 eq.) was used instead of phenyl boronic acid pinacol ester. Purification by flash chromatography on silica gel, eluting with 1:2 light petroleum/ethyl acetate → ethyl acetate gave the *title compound* **11** (28 mg, 90%) as an orange solid; TLC R_f = 0.38 (ethyl acetate, det: $KMnO_4$/Δ); mp 116-118 °C; $[a]_D^{22}$ +338.7 (*c* 0.34, $CHCl_3$); (Found: M+Na^+, 782.3294. $C_{41}H_{49}N_3O_{11}$+Na^+, requires 782.3259); ν_{max} ($CHCl_3$)/cm^{-1} 3545, 3431, 3004, 1712, 1663, 1586, 1397, 1240, 1054, 1029; δ_H (500 MHz; DMSO-d_6) 9.66 (1H, s, NH), 7.89-7.87 (2H, m, H-35), 7.85-7.83 (2H, m, H-36), 6.75 (1H, dt, *J* 15.9, 7.1, H-30), 6.47 (1H, dt, *J* 15.9, 2.0, H-29), 6.42-6.22 (2H, br. s, NH_2), 6.36-6.29 (2H, m, H-3+4), 5.22 (1H, t, *J* 10.0, H-5), 5.14 (1H, d, *J* 10.3, H-9), 4.85 (1H, d, *J* 9.3, H-7), 4.38 (1H, d, *J* 4.2, OH), 3.95 (1H, dd, *J* 10.0, 9.3, H-6), 3.92 (3H, s, OMe-26), 3.82-3.72 (2H, m, H-32), 3.46 (1H, ddd, *J* 9.6, 4.2, 2.7, H-11), 3.18 (3H, s, OMe-28), 3.04 (3H, s, OMe-27), 2.78 (1H, dt, *J* 9.0, 2.7, H-12), 2.61 (2H, tdd, *J* 7.3, 7.1, 2.0, H-31), 2.45 (1H, dd, *J* 12.4, 5.9, H-15), 2.33 (1H, dd, *J* 12.4, 4.4, H-15), 2.12-2.02 (2H, m, H-10+14), 1.84 (3H, s, Me-22), 1.39 (1H, ddd, *J* 13.9, 9.0, 4.0, H-13), 1.19 (3H, s, Me-23), 0.87 (3H, d, *J* 6.5, Me-24), 0.64 (1H, ddd, *J* 13.9, 11.6, 2.7, H-13), 0.60 (3H, d, *J* 6.7, Me-25); δ_C (125 MHz; DMSO-d_6) 183.7 (C=O-21), 181.7 (C=O-18), 173.4 (C=O-1), 167.8 (C=O-33), 156.4 (C-17), 155.7 (OC=ONH$_2$), 138.4 (C-2), 138.0 (CH-30), 137.6 (C-20), 134.3 (CH-36), 134.2 (CH-9), 131.6 (C-34), 130.3 (CH-5), 128.5 (C-16), 128.5 (C-8), 128.2 (CH-4), 124.5 (C-19), 123.1 (CH-3), 123.0 (CH-35), 121.4 (CH-29), 80.0 (CH-7), 79.5 (CH-12), 74.4 (CH-6), 71.5 (CH-11), 60.8 (Me-26), 55.6 (Me-28), 55.4 (Me-27), 36.7 (CH_2-32), 34.7 (CH-10), 33.0 (CH_2-31), 30.5 (CH_2-13), 29.5 (CH_2-15), 28.4 (CH-14), 18.8 (Me-25), 18.5 (Me-24), 13.8 (Me-22), 11.3 (Me-23); *m/z* (ESI) 782 ($[M+Na]^+$, 100%).

(4E,6Z,8S,9S,10E,12S,13R,14S,16R)-13-Hydroxy-21-(4-hydroxybutanoyl)-8,14,19-trimethoxy-4,10,12,16-tetramethyl-3,20,22-trioxo-2-azabicyclo[16.3.1]docosa-1(21),4,6,10,18-pentaen-9-yl carbamate

***[19-(4-Hydroxybutanoyl)geldanamycin]* 12**

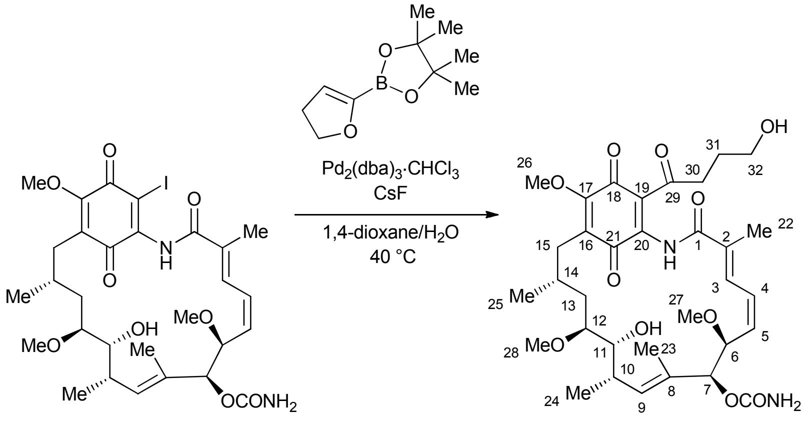

The reaction was carried out according to general procedure 1, using 19-iodogeldanamycin **4**[5] (29 mg, 0.042 mmol, 1.0 eq.), *tris*-(dibenzylideneacetone)dipalladium(0) chloroform complex (2 mg, 0.002 mmol, 5 mol%) and caesium fluoride (13 mg, 0.085 mmol, 2.0 eq.) in 1,4-dioxane/water (9:1, 2 mL), differing only in that 2,3-dihydro-5-furylboronic acid pinacol ester (17 mg, 0.085 mmol, 2.0 eq.) was used instead of phenyl boronic acid pinacol ester. Purification by flash chromatography on silica gel, eluting with 1:2 light petroleum/ethyl acetate → ethyl acetate gave the *title compound* **12** (14 mg, 53%) as a yellow solid; TLC R_f = 0.20 (ethyl acetate, det: $KMnO_4$/Δ); mp 132-134 °C; $[a]_D^{22}$ +730.2 (*c* 0.01, $CHCl_3$); (Found: M+Na$^+$, 669.2972. $C_{33}H_{46}N_2O_{11}$+Na$^+$, requires 669.2994); ν_{max} ($CHCl_3$)/cm^{-1} 3652, 3431, 3001 (br.), 1729, 1658, 1583, 1444, 1370, 1054, 1030; δ_H (500 MHz; DMSO-d_6) 9.97 (1H, s, NH), 7.92 (2H, br. s, 32-OH), 6.77-6.16 (2H, br. s, NH_2), 6.67 (1H, d, *J* 10.7, H-3), 6.39 (1H, t, *J* 10.7, H-4), 5.35 (1H, dd, *J* 10.7, 8.8, H-5), 5.18 (1H, d, *J* 10.4, H-9), 4.89 (1H, d, *J* 8.8, H-

7), 4.34 (1H, d, *J* 4.2, 11-OH), 4.12 (1H, t, *J* 8.8, H-6), 3.98 (3H, s, OMe-26), 3.48-3.42 (3H, m, H-11+32), 3.18 (3H, s, OMe-28), 3.08 (3H, s, OMe-27), 2.86 (2H, t, *J* 7.9, H-30), 2.83-2.76 (1H, m, H-12), 2.48 (1H, dd, *J* 12.5, 5.7, H-15), 2.36 (1H, dd, *J* 12.5, 4.7, H-15), 2.17-2.09 (1H, m, H-10), 2.09-2.01 (1H, m, H-14), 1.83 (3H, s, Me-22), 1.78 (2H, quintet, *J* 7.9, H-31), 1.42 (1H, ddd, *J* 13.7, 8.7, 4.3, H-13), 1.21 (3H, s, Me-23), 0.87 (3H, d, *J* 6.5, H-24), 0.76-0.67 (1H, m, H-13), 0.61 (3H, d, *J* 6.2, H-25); δ_C (125 MHz; DMSO-d_6) 202.0 (C=O-29), 183.7 (C=O-21), 180.9 (C=O-18), 174.5 (C=O-1), 156.1 (C-17), 155.8 (OC=ONH_2), 139.0 (C-20), 138.5 (C-2), 133.6 (CH-9), 129.1 (CH-5), 128.5 (C-8), 127.9 (CH-4), 126.5 (C-16), 125.3 (CH-3), 121.7 (C-19), 79.8 (CH-7), 79.6 (CH-12), 74.9 (CH-6), 71.4 (CH-11), 61.1 (Me-26), 60.1 (CH_2-32), 55.6 (Me-28), 55.6 (Me-27), 40.8 (CH_2-30), 34.5 (CH-10), 29.7 (CH_2-15), 28.9 (CH_2-13), 28.5 (CH-14), 26.2 (CH_2-31), 19.1 (Me-25), 18.2 (Me-24), 13.4 (Me-22), 11.7 (Me-23); *m/z* (ESI) 669 ($[M+Na]^+$, 100%).

(4E,6Z,8S,9S,10E,12S,13R,14S,16R)-21-(3,4-Dihydro-2H-pyran-6-yl)-13-hydroxy-8,14,19-trimethoxy-4,10,12,16-tetramethyl-3,20,22-trioxo-2-azabicyclo[16.3.1]docosa-1(21),4,6,10,18-pentaen-9-yl carbamate

***[19-(3,4-Dihydro-2H-pyran-6-yl)geldanamycin]* 13**

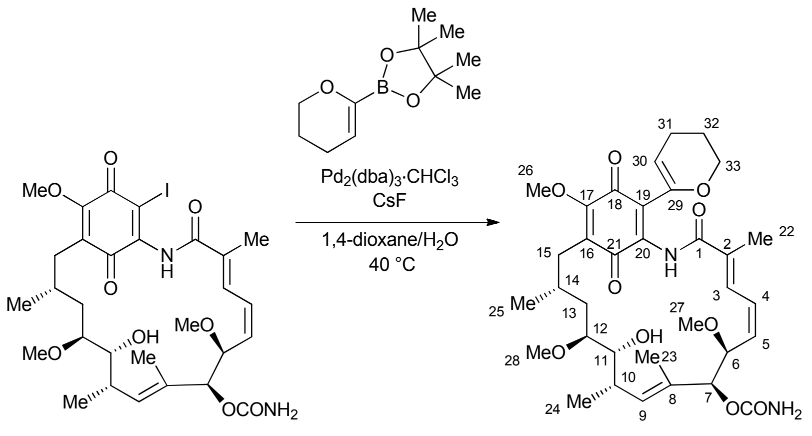

4 13

This journal is © The Royal Society of Chemistry 2013

The reaction was carried out according to general procedure 1, using 19-iodogeldanamycin **4**[5] (30 mg, 0.044 mmol, 1.0 eq.), *tris*-(dibenzylideneacetone)dipalladium(0) chloroform complex (2.5 mg, 0.002 mmol, 5 mol%) and caesium fluoride (13 mg, 0.087 mmol, 2.0 eq.) in 1,4-dioxane/water (9:1, 2 mL), differing only in that 3,4-dihydro-2*H*-pyran-6-boronic acid pinacol ester (18 mg, 0.087 mmol, 2.0 eq.) was used instead of phenyl boronic acid pinacol ester. Purification by flash chromatography on silica gel, eluting with 1:2 light petroleum/ethyl acetate → ethyl acetate gave the *title compound* **13** (13 mg, 46%) as an orange solid; TLC R_f = 0.30 (ethyl acetate, det: $KMnO_4$/Δ); mp 128-130 °C; $[a]_D^{22}$ −13.5 (*c* 0.13, $CHCl_3$); (Found: $M+Na^+$, 660.3474. $C_{34}H_{46}N_2O_{10}+NH_4^+$, requires 660.9491); ν_{max} ($CHCl_3$)/cm^{-1} 3543, 3432, 3045, 1731, 1661, 1585, 1368, 1242, 1054; δ_H (500 MHz; DMSO-d_6) 9.55 (1H, s, NH), 6.60-6.17 (2H, br. s, NH_2), 6.59 (1H, d, *J* 11.9, H-3), 6.33 (1H, dd, *J* 11.9, 10.8, H-4), 5.24 (1H, t, *J* 10.8, H-5), 5.12 (1H, d, *J* 9.7, H-9), 5.03 (1H, t, *J* 3.9, H-30), 4.84 (1H, d, *J* 9.4, H-7), 4.34 (1H, br. s, OH), 4.15-4.08 (2H, m, H-33), 3.95 (1H, dd, *J* 10.8, 9.4, H-6), 3.93 (3H, s, OMe-26), 3.45 (1H, dd, *J* 9.6, 1.8, H-11), 3.18 (3H, s, H-28), 3.03 (3H, s, H-27), 2.79 (1H, dt, *J* 9.6, 3.1, H-12), 2.46 (1H, dd, *J* 12.4, 5.8, H-15), 2.36 (1H, dd, *J* 12.4, 4.3, H-15), 2.19-2.15 (2H, m, H-31), 2.12-2.03 (2H, m, H-10+14), 1.94-1.87 (2H, m, H-32), 1.81 (3H, s, Me-22), 1.44-1.36 (1H, m, H-13), 1.22 (3H, s, Me-23), 0.87 (3H, d, *J* 6.5, H-24), 0.61 (1H, td, *J* 14.6, 3.1, H-13), 0.57 (3H, d, *J* 6.8, H-25); δ_C (125 MHz; DMSO-d_6) 184.6 (C=O-21), 180.8 (C=O-18), 173.5 (C=O-1), 156.8 (C-17), 156.3 ($OC=ONH_2$), 143.5 (C-20), 140.4 (C-29), 139.3 (C-2), 134.2 (CH-9), 131.1 (CH-5), 129.3 (C-8), 129.1 (C-16), 128.7 (CH-4), 124.9 (C-19), 124.8 (CH-3), 107.4 (CH-30), 80.5 (CH-7), 80.1 (CH-12), 75.1 (CH-6), 72.0 (CH-11), 66.5 (CH_2-33), 61.4 (Me-26), 56.3 (Me-27), 56.1 (Me-28), 35.3 (CH-10), 30.8 (CH_2-13), 29.9 (CH_2-15), 29.1 (CH-14), 22.1 (CH_2-32), 20.7 (CH_2-31), 19.2 (Me-24), 19.1 (Me-25), 14.1 (Me-22), 12.2 (Me-23); *m/z* (ESI) 665 ($[M+Na]^+$, 100%).

(4E,6Z,8S,9S,10E,12S,13R,14S,16R)-13-Hydroxy-8,14,19-trimethoxy-4,10,12,16-tetramethyl-3,20,22-trioxo-21-(p-tolyl)-2-azabicyclo[16.3.1]docosa-1(21),4,6,10,18-pentaen-9-yl carbamate

***[19-(p-Tolyl)geldanamycin]* 14**

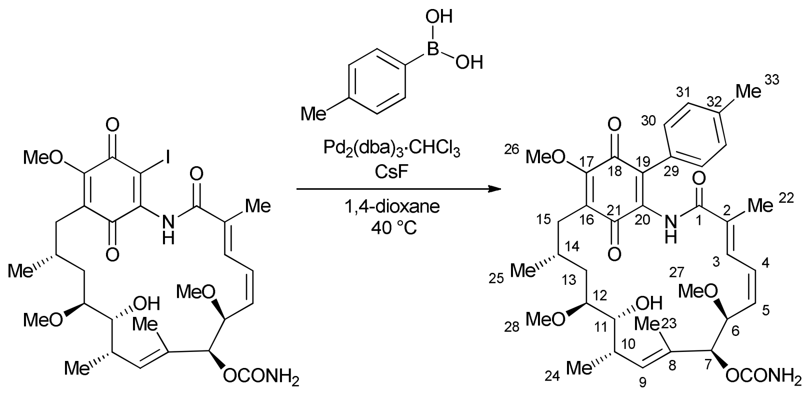

The reaction was carried out according to general procedure 1, using 19-iodogeldanamycin **4**[5] (49 mg, 0.071 mmol, 1.0 eq.), *tris*-(dibenzylideneacetone)dipalladium(0) chloroform complex (4 mg, 0.004 mmol, 5 mol%) and caesium fluoride (22 mg, 0.143 mmol, 2.0 eq.), differing only in that 4-methylbenzeneboronic acid (19 mg, 0.143 mmol, 2.0 eq.) was used instead of phenyl boronic acid pinacol ester and the reaction was performed in 1,4-dioxane (2 mL) instead of 1,4-dioxane/water. Purification by flash chromatography on silica gel, eluting with 1:2 light petroleum/ethyl acetate → ethyl acetate gave the *title compound* **14** (49 mg, quantitative yield) as an orange solid; TLC R_f = 0.23 (1:2 light petroleum/ethyl acetate, det: $KMnO_4/\Delta$); mp 101-102 °C; $[a]_D^{22}$ +113.3 (*c* 1.0, $CHCl_3$); (Found: $M+Na^+$, 673.3060. $C_{36}H_{46}N_2O_9+Na^+$, requires 673.3096); ν_{max} ($CHCl_3$)/cm^{-1} 3547, 3432, 3358, 3004, 1731, 1712, 1656, 1592, 1367, 1290, 1240, 1053; δ_H (500 MHz; DMSO-d_6) 9.45 (1H, s, NH), 7.34 (2H, d, *J* 8.0, H-31), 7.28 (2H, d, *J* 8.0, H-30), 6.59-6.14 (2H, br. s, NH_2), 6.53 (1H, d, *J* 11.8, H-3), 6.41 (1H, dd, *J* 11.8, 10.7, H-4), 5.31 (1H, dd, *J* 10.7, 9.9, H-5), 5.17 (1H, d, *J* 10.4, H-9),

4.90 (1H, d, *J* 9.9, H-7), 4.37 (1H, d, *J* 4.2, OH), 3.98 (1H, t, *J* 9.9, H-6), 3.96 (3H, s, OMe-26), 3.47 (1H, ddd, *J* 9.4, 4.2, 2.9, H-11), 3.20 (3H, s, OMe-28), 3.10 (3H, s, OMe-27), 2.82 (1H, dt, *J* 9.1, 2.9, H-12), 2.54 (1H, dd, *J* 12.4, 5.9, H-15), 2.42 (1H, dd, *J* 12.4, 4.4, H-15), 2.38 (3H, s, Me-33), 2.16-2.04 (2H, m, H-10+14), 1.85 (3H, s, Me-22), 1.44 (1H, ddd, *J* 13.9, 9.1, 4.1, H-13), 1.22 (3H, s, Me-23), 0.87 (3H, d, *J* 6.5, Me-24), 0.70 (1H, td, *J* 13.9, 2.9, H-13), 0.65 (3H, d, *J* 6.7, Me-25); δ_C (125 MHz; DMSO-d_6) 184.1 (C=O-21), 181.4 (C=O-18), 172.7 (C=O-1), 156.6 (C-17), 155.7 (OC=ONH$_2$), 139.5 (C-20), 138.7 (C-2), 137.9 (C-32), 134.1 (CH-9), 130.3 (CH-5), 129.7 (C-19), 129.6 (CH-30), 128.7 (CH-31), 128.5 (C-16), 128.5 (C-8), 128.1 (C-29), 128.0 (CH-4), 122.7 (CH-3), 79.8 (CH-7), 79.6 (CH-12), 74.8 (CH-6), 71.4 (CH-11), 60.9 (Me-26), 55.8 (Me-27), 55.6 (Me-28), 34.7 (CH-10), 30.6 (CH$_2$-13), 29.6 (CH$_2$-15), 28.6 (CH-14), 20.8 (Me-33), 18.8 (Me-25), 18.5 (Me-24), 13.8 (Me-22), 11.6 (Me-23); *m/z* (ESI) 673 ([M+Na]$^+$, 100%).

(4E,6Z,8S,9S,10E,12S,13R,14S,16R)-13-Hydroxy-8,14,19-trimethoxy-21-(4-methoxyphenyl)-4,10,12,16-tetramethyl-3,20,22-trioxo-2-azabicyclo[16.3.1]docosa-1(21),4,6,10,18-pentaen-9-yl carbamate

***[19-(4-Methoxyphenyl)geldanamycin]* 15**

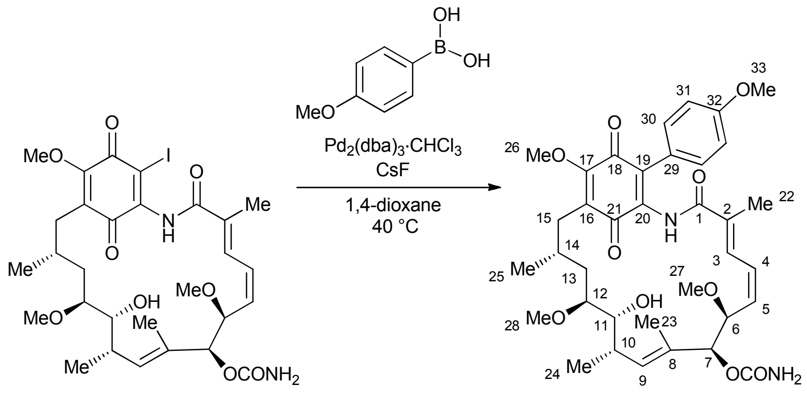

4 15

The reaction was carried out according to general procedure 1, using 19-iodogeldanamycin **4**[5] (27 mg, 0.039 mmol, 1.0 eq.), *tris*-(dibenzylideneacetone)dipalladium(0) chloroform complex (2 mg, 0.002 mmol, 5 mol%) and caesium fluoride (12 mg, 0.079 mmol, 2.0 eq.), differing only in that 4-methoxyphenylboronic acid (12 mg, 0.079 mmol, 2.0 eq.) was used instead of phenyl boronic acid pinacol ester and 1,4-dioxane (2 mL) was used instead of 1,4-dioxane/water. Purification by flash chromatography on silica gel, eluting with ethyl acetate gave the *title compound* **15** (25 mg, 95%) as an orange solid; TLC R_f = 0.35 (ethyl acetate, det: $KMnO_4$/Δ); mp 136-137 °C; $[a]_D^{22}$ +340.3 (*c* 0.2, $CHCl_3$); δ_H (500 MHz; DMSO-d_6) 9.48 (1H, s, NH), 7.34 (2H, d, *J* 8.9, H-30), 7.10 (2H, d, *J* 8.9, H-31), 6.51 (1H, d, *J* 11.7, H-3), 6.54-6.20 (2H, br. s, NH_2), 6.41 (1H, dd, *J* 11.7, 10.7, H-4), 5.29 (1H, t, *J* 10.7, H-5), 5.16 (1H, d, *J* 10.4, H-9), 4.89 (1H, d, *J* 10.7, H-7), 4.40 (1H, d, *J* 4.2, OH), 3.96 (1H, t, *J* 10.7, H-6), 3.96 (3H, s, OMe-26), 3.83 (3H, s, OMe-33), 3.47 (1H, ddd, *J* 8.7, 4.2, 2.5, H-11), 3.20 (3H, s, OMe-28), 3.10 (3H, s, OMe-27), 2.81 (1H, dt, *J* 9.4, 2.5, H-12), 2.54 (1H, dd, *J* 12.4, 5.9, H-15), 2.40 (1H, dd, *J* 12.4, 4.4, H-15), 2.13-2.04 (2H, m, H-10+14), 1.86 (3H, s, Me-22), 1.44 (1H, ddd, *J* 13.8, 9.4, 4.0, H-13), 1.20 (3H, s, Me-23), 0.86 (3H, d, *J* 6.5, Me-24), 0.69 (1H, dd, *J* 13.8, 10.8, 2.5, H-13), 0.64 (3H, d, *J* 6.7, Me-25). Data are consistent with those reported in the literature.[6]

(4E,6Z,8S,9S,10E,12S,13R,14S,16R)-13-Hydroxy-21-(4-hydroxyphenyl)-8,14,19-trimethoxy-4,10,12,16-tetramethyl-3,20,22-trioxo-2-azabicyclo[16.3.1]docosa-1(21),4,6,10,18-pentaen-9-yl carbamate

***[19-(4-Hydroxyphenyl)geldanamycin]* 16**

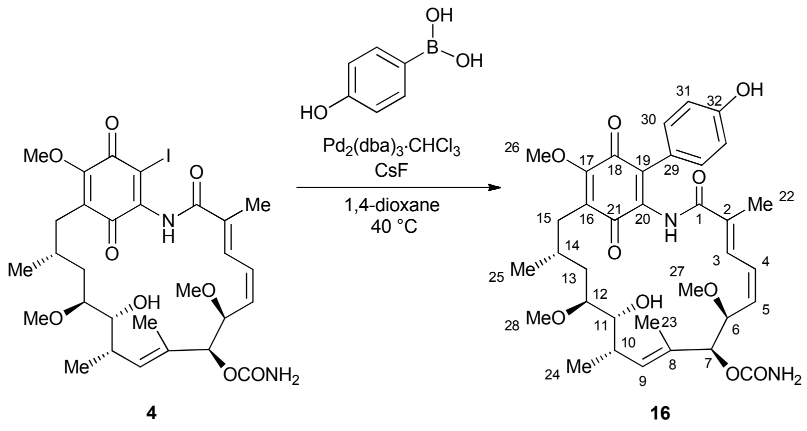

The reaction was carried out according to general procedure 1, using 19-iodogeldanamycin **4**[5] (56 mg, 0.082 mmol, 1.0 eq.), *tris*-(dibenzylideneacetone)dipalladium(0) chloroform complex (4 mg, 0.004 mmol, 5 mol%) and caesium fluoride (25 mg, 0.163 mmol, 2.0 eq.), differing only in that 4-hydroxybenzeneboronic acid (23 mg, 0.163 mmol, 2.0 eq.) was used instead of phenyl boronic acid pinacol ester and the reaction was performed in 1,4-dioxane (2 mL) instead of 1,4-dioxane/water. Purification by flash chromatography on silica gel, eluting with 1:2 light petroleum/ethyl acetate → ethyl acetate gave the *title compound* **16** (43 mg, 81%) as a brown solid; TLC R_f = 0.09 (1:2 light petroleum/ethyl acetate, det: $KMnO_4$/Δ); mp 117-118 °C; $[a]_D^{22}$ +236.7 (*c* 0.33, $CHCl_3$); (Found: M+Na$^+$, 675.2848. $C_{35}H_{44}N_2O_{10}$+Na$^+$, requires 675.2888); ν_{max} ($CHCl_3$)/cm^{-1} 3694, 3455, 2999, 1708, 1651, 1592, 1239, 1054; δ_H (500 MHz; DMSO-*d*$_6$) 9.78 (1H, s, 32-OH), 9.39 (1H, s, NH), 7.24 (2H, d, *J* 8.7, H-30), 6.89 (2H, d, *J* 8.7, H-31), 6.62-6.12 (2H, br. s, NH_2), 6.51 (1H, d, *J* 11.5, H-3), 6.40 (1H, dd, *J* 11.5, 10.7, H-4), 5.29 (1H, dd, *J* 10.7, 9.5, H-5), 5.18 (1H, d, *J* 10.4, H-9), 4.90 (1H, d, *J* 9.5, H-7), 4.37 (1H, d, *J* 4.2, 11-OH), 3.95 (1H, t, *J* 9.5, H-6), 3.95 (3H, s, OMe-26), 3.47 (1H, ddd, *J* 9.4, 4.2, 3.3, H-11), 3.20 (3H, s, OMe-28), 3.10 (3H, s, OMe-27), 2.81 (1H, dt, *J* 9.1, 3.3, H-12), 2.53 (1H, dd, *J* 12.5, 5.9, H-15), 2.40 (1H, dd, *J* 12.5, 4.4, H-15), 2.15-2.02 (2H, m, H-10+14), 1.86 (3H, s, Me-22), 1.43 (1H, ddd, *J* 13.9, 9.1, 4.1, H-13), 1.20 (3H, s, Me-23), 0.87 (3H, d, *J* 6.5, Me-24), 0.70 (1H, td, *J* 13.9, 3.3, H-13), 0.64 (3H, d, *J* 6.7, Me-25); δ_C (125 MHz;

DMSO-d_6) 184.1 (C=O-21), 181.7 (C=O-18), 172.7 (C=O-1), 157.7 (C-32), 156.5 (C-17), 155.7 (OC=ONH$_2$), 138.7 (C-2), 138.6 (C-20), 134.1 (CH-9), 131.3 (CH-30), 130.2 (CH-5), 128.6 (C-16), 128.5 (C-19), 128.5 (C-8), 128.2 (CH-4), 122.5 (CH-3), 121.2 (C-29), 114.9 (CH-31), 79.8 (CH-7), 79.6 (CH-12), 74.7 (CH-6), 71.4 (CH-11), 60.8 (Me-26), 55.8 (Me-27), 55.6 (Me-28), 34.7 (CH-10), 30.6 (CH$_2$-13), 29.6 (CH$_2$-15), 28.6 (CH-14), 18.8 (Me-25), 18.5 (Me-24), 13.8 (Me-22), 11.6 (Me-23); *m/z* (ESI) 675 ([M+Na]$^+$, 100%).

(4E,6Z,8S,9S,10E,12S,13R,14S,16R)-13-Hydroxy-8,14,19-trimethoxy-4,10,12,16-tetramethyl-21-(2-nitrophenyl)-3,20,22-trioxo-2-azabicyclo[16.3.1]docosa-1(21),4,6,10,18-pentaen-9-yl carbamate

***[19-(2-Nitrophenyl)geldanamycin]* 17**

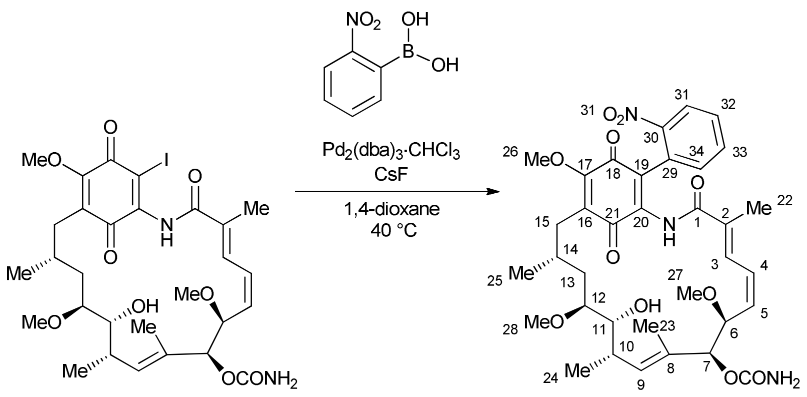

4 **17**

The reaction was carried out according to general procedure 1, using 19-iodogeldanamycin **4**[5] (47 mg, 0.069 mmol, 1.0 eq.), *tris*-(dibenzylideneacetone)dipalladium(0) chloroform complex (4 mg, 0.003 mmol, 5 mol%) and caesium fluoride (21 mg, 0.137 mmol, 2.0 eq.), differing only in that 2-nitrophenylboronic acid (23 mg, 0.137 mmol, 2.0 eq.) was used instead of phenyl boronic acid pinacol ester and the reaction was performed in 1,4-dioxane (2 mL) instead of 1,4-dioxane/water. Purification by flash chromatography on silica gel, eluting with

1:2 light petroleum/ethyl acetate → ethyl acetate gave the *title compound* **17** (30 mg, 64%) as a dark yellow solid; TLC R_f = 0.21 (1:2 light petroleum/ethyl acetate, det: $KMnO_4/\Delta$); mp 102-104 °C; $[a]_D^{22}$ +26.1 (*c* 0.05, $CHCl_3$); (Found: $M+Na^+$, 704.2768. $C_{35}H_{43}N_3O_{11}+Na^+$, requires 704.2790); ν_{max} ($CHCl_3$)/cm^{-1} 3697, 3546, 3431, 2993, 2932, 1731, 1657, 1605, 1349, 1054; δ_H (500 MHz; DMSO-d_6) 9.90 (1H, s, NH), 8.38 (1H, dd, *J* 8.5, 1.1, H-31), 8.02 (1H, ddd, *J* 8.5, 7.6, 1.1, H-33), 7.81 (1H, td, *J* 8.5, 1.3, H-32), 7.49 (1H, dd, *J* 7.6, 1.3, H-34), 6.64-6.14 (2H, br. s, NH_2), 6.58 (1H, d, *J* 11.2, H-3), 6.43 (1H, dd, *J* 11.2, 10.8, H-4), 5.35 (1H, dd, *J* 10.8, 9.7, H-5), 5.21 (1H, d, *J* 10.5, H-9), 4.93 (1H, d, *J* 9.7, H-7), 4.42 (1H, d, *J* 4.3, OH), 4.09 (1H, t, *J* 9.7, H-6), 3.90 (3H, s, OMe-26), 3.50 (1H, ddd, *J* 9.7, 4.3, 2.9, H-11), 3.21 (3H, s, OMe-28), 3.16 (3H, s, OMe-27), 2.83 (1H, dt, *J* 9.1, 2.9, H-12), 2.58 (1H, dd, *J* 12.4, 5.7, H-15), 2.47 (1H, dd, *J* 12.4, 4.5, H-15), 2.18-2.04 (2H, m, H-10+14), 1.88 (3H, s, Me-22), 1.46 (1H, ddd, *J* 13.9, 9.1, 4.1, H-13), 1.27 (3H, s, Me-23), 0.87 (3H, d, *J* 6.4, Me-24), 0.81-0.76 (1H, m, H-13), 0.66 (3H, d, *J* 6.7, Me-25); δ_C (125 MHz; DMSO-d_6) 183.5 (C=O-21), 180.3 (C=O-18), 172.8 (C=O-1), 156.5 (C-17), 155.7 (OC=ONH_2), 148.0 (C-30), 139.4 (C-2), 139.0 (C-20), 134.9 (CH-33), 134.4 (CH-9), 132.9 (CH-5), 130.5 (C-29), 129.9 (CH-32), 129.8 (C-16), 129.8 (CH-34), 128.2 (C-8), 128.1 (CH-4), 127.6 (C-19), 125.2 (CH-31), 123.2 (CH-3), 79.8 (CH-7), 79.4 (CH-12), 74.9 (CH-6), 71.3 (CH-11), 61.1 (Me-26), 55.8 (Me-27), 55.6 (Me-28), 34.8 (CH-10), 31.1 (CH_2-13), 29.7 (CH_2-15), 28.5 (CH-14), 18.9 (Me-25), 18.4 (Me-24), 13.9 (Me-22), 11.5 (Me-23); *m/z* (ESI) 704 ($[M+Na]^+$, 100%).

(4E,6Z,8S,9S,10E,12S,13R,14S,16R)-21-(4-Acetylphenyl)-13-hydroxy-8,14,19-trimethoxy-4,10,12,16-tetramethyl-3,20,22-trioxo-2-azabicyclo[16.3.1]docosa-1(21),4,6,10,18-pentaen-9-yl carbamate

[19-(4-Acetylphenyl)geldanamycin] **18**

Electronic Supplementary Material (ESI) for Chemical Communications
This journal is © The Royal Society of Chemistry 2013

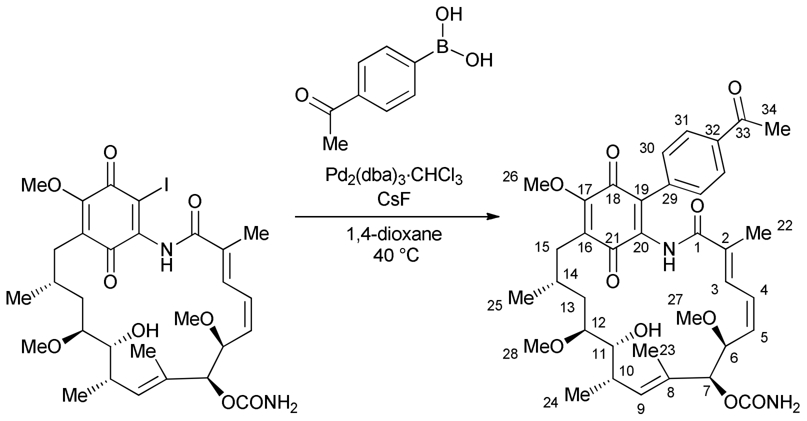

4 **18**

The reaction was carried out according to general procedure 1, using 19-iodogeldanamycin **4**[5] (48 mg, 0.070 mmol, 1.0 eq.), *tris*-(dibenzylideneacetone)dipalladium(0) chloroform complex (4 mg, 0.004 mmol, 5 mol%) and caesium fluoride (21 mg, 0.140 mmol, 2.0 eq.), differing only in that 4-acetylbenzeneboronic acid (23 mg, 0.140 mmol, 2.0 eq.) was used instead of phenyl boronic acid pinacol ester and the reaction was performed in 1,4-dioxane (2 mL) instead of 1,4-dioxane/water. Purification by flash chromatography on silica gel, eluting with 1:2 light petroleum/ethyl acetate → ethyl acetate gave the *title compound* **18** (31 mg, 65%) as an orange solid; TLC R_f = 0.12 (1:2 light petroleum/ethyl acetate, det: $KMnO_4/\Delta$); mp 114-116 °C; $[a]_D^{22}$ +76.5 (*c* 0.61, $CHCl_3$); (Found: $M+Na^+$, 701.3019. $C_{37}H_{46}N_2O_{10}+Na^+$, requires 701.3045); ν_{max} ($CHCl_3$)/cm^{-1} 3692, 3546, 3431, 3004, 2934, 1732, 1685, 1655, 1365, 1267, 1054; δ_H (500 MHz; DMSO-d_6) 9.65 (1H, s, NH), 8.09 (2H, d, *J* 8.5, H-31), 7.52 (2H, d, *J* 8.5, H-30), 6.56 (1H, d, *J* 11.6, H-3), 6.50-6.15 (2H, br. s, NH_2), 6.42 (1H, t, *J* 11.6, 10.7, H-4), 5.33 (1H, t, *J* 10.7, H-5), 5.19 (1H, d, *J* 10.4, H-9), 4.90 (1H, d, *J* 9.7, H-7), 4.38 (1H, d, *J* 4.1, OH), 4.03 (1H, dd, *J* 10.7, 9.7, H-6), 3.98 (3H, s, OMe-26), 3.47 (1H, ddd, *J* 9.4, 4.1, 3.0, H-11), 3.20 (3H, s, OMe-28), 3.13 (3H, s, OMe-27), 2.83 (1H, dt, *J* 9.0, 3.0, H-12), 2.65 (3H, s, Me-34), 2.55 (1H, dd, *J* 12.4, 5.9, H-15), 2.42 (1H, dd, *J* 12.4, 4.5, H-15), 2.16-2.03 (2H, m, H-10+14), 1.86 (3H, s, Me-22), 1.45 (1H, ddd, *J* 13.9, 9.0, 4.1, H-13), 1.23 (3H, s, Me-23),

0.86 (3H, d, *J* 6.5, Me-24), 0.72 (1H, td, *J* 13.9, 3.0, H-13), 0.66 (3H, d, *J* 6.7, Me-25); δ_C (125 MHz; DMSO-d_6) 197.6 (C=O-33), 183.9 (C=O-21), 181.0 (C=O-18), 172.7 (C=O-1), 156.6 (C-17), 155.7 (OC=ONH$_2$), 140.1 (C-20), 138.8 (C-2), 136.3 (C-32), 135.9 (C-29), 134.2 (CH-9), 130.6 (CH-5), 130.1 (CH-30), 128.7 (C-16), 128.4 (C-8), 128.2 (C-19), 128.1 (CH-4), 127.9 (CH-31), 123.0 (CH-3), 79.8 (CH-7), 79.6 (CH-12), 74.9 (CH-6), 71.4 (CH-11), 61.0 (Me-26), 55.8 (Me-27), 55.6 (Me-28), 34.7 (CH-10), 30.6 (CH$_2$-13), 29.7 (CH$_2$-15), 28.5 (CH-14), 26.8 (Me-34), 18.9 (Me-25), 18.4 (Me-24), 13.8 (Me-22), 11.5 (Me-23); *m/z* (ESI) 701 ([M+Na]$^+$, 100%).

(4E,6Z,8S,9S,10E,12S,13R,14S,16R)-13-Hydroxy-8,14,19-trimethoxy-4,10,12,16-tetramethyl-3,20,22-trioxo-21-(2-thienyl)-2-azabicyclo[16.3.1]docosa-1(21),4,6,10,18-pentaen-9-yl carbamate

***[19-(2-Thienyl)geldanamycin]* 19**

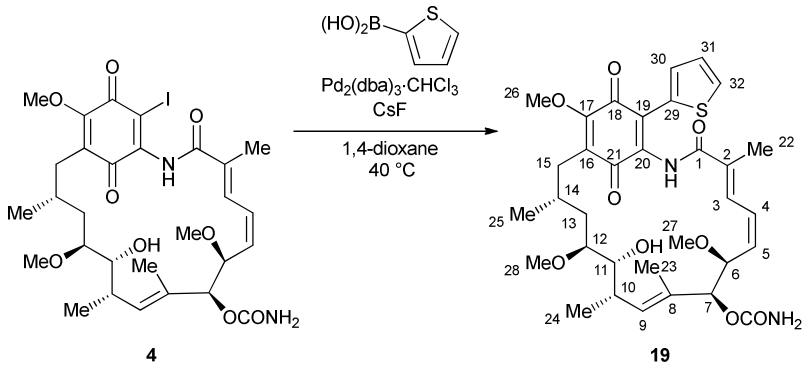

The reaction was carried out according to general procedure 1, using 19-iodogeldanamycin **4**[5] (25 mg, 0.036 mmol, 1.0 eq.), *tris*-(dibenzylideneacetone)dipalladium(0) chloroform complex (2 mg, 0.002 mmol, 5 mol%) and caesium fluoride (11 mg, 0.073 mmol, 2.0 eq.), differing only in that thiophen-2-boronic acid (9 mg, 0.073 mmol, 2.0 eq.) was used instead of phenyl boronic acid pinacol ester and 1,4-dioxane (2 mL) was used instead of 1,4-dioxane/water.

This journal is © The Royal Society of Chemistry 2013

Purification by flash chromatography on silica gel, eluting with 1:2 light petroleum/ethyl acetate → ethyl acetate gave the *title compound* **19** (17 mg, 73%) as a red solid; TLC R_f = 0.64 (ethyl acetate, det: $KMnO_4/\Delta$); mp 242-243 °C; $[a]_D^{24}$ +958.0 (*c* 0.03, $CHCl_3$); δ_H (500 MHz; DMSO-d_6) 9.83 (1H, s, NH), 7.91 (1H, dd, *J* 5.0, 0.7, H-32), 7.77 (1H, dd, *J* 3.9, 0.7, H-30), 7.28 (1H, dd, *J* 5.0, 3.9, H-31), 6.46 (1H, d, *J* 11.7, H-3), 6.43-6.19 (2H, br. s, NH_2), 6.39 (1H, dd, *J* 11.0, 10.5, H-5), 5.23 (1H, dd, *J* 11.7, 10.5, H-4), 5.14 (1H, d, *J* 10.5, H-9), 4.84 (1H, d, *J* 9.4, H-7), 4.42 (1H, d, *J* 4.2, OH), 3.98 (3H, s, OMe-26), 3.78 (1H, dd, *J* 11.0, 9.4, H-6), 3.46 (1H, ddd, *J* 9.5, 4.2, 2.8, H-11), 3.19 (3H, s, H-28), 3.01 (3H, s, H-27), 2.77 (1H, dt, *J* 9.4, 2.8, H-12), 2.52 (1H, dd, *J* 12.4, 5.9, H-15), 2.40 (1H, dd, *J* 12.4, 4.3, H-15), 2.11-2.04 (2H, m, H-10+14), 1.87 (3H, s, Me-22), 1.44 (1H, ddd, *J* 14.1, 9.4, 4.1, H-13), 1.10 (3H, s, Me-23), 0.85 (3H, d, *J* 6.5, H-24), 0.64 (1H, dd, *J* 14.1, 11.7, 2.8, H-13), 0.60 (3H, d, *J* 6.7, H-25). Data are consistent with those reported in the literature.[6]

References

1. Knapp, D. M., Gillis, E. P., Burke, M. D., *J. Am. Chem. Soc.*, 2009, **131**, 6961-6963.
2. Molander, G. A., Katona, B. W., Machrouhi, F., *J. Org. Chem.*, 2002, **67**, 8416-8423.
3. Iyer, S., Liebeskind, L. S., *J. Am. Chem. Soc.*, 1987, **109**, 2759-2770.
4. Shade, R. E., Hyde, A. M., Olsen, J.-C., Merlic, C. A., *J. Am. Chem. Soc.*, 2013, **132**, 1202-1203.
5. Sasaki, K., Inoue, Y., 1980, German Patent No. Ger. Offen. 3006097.
6. Kitson, R. R. A., Chuan, C.-H., Xiong, R., Williams, H. E. L., Davis, A. L., Lewis, W., Dehn, D. L., Siegel, D., Roe, S. M., Prodromou, C., Ross, D., Moody, C. J., *Nature Chem.*, 2013, **5**, 307-314.
