# Supplementary figures and images for: An improved route to 19-substituted geldanamycins as novel Hsp90 inhibitors – potential therapeutics in cancer and neurodegeneration
Source: Chem Commun (Camb). 2013 Jun 17;49(76):8441–3. doi: 10.1039/c3cc43457e (PMC3835074; doi:10.1039/c3cc43457e)

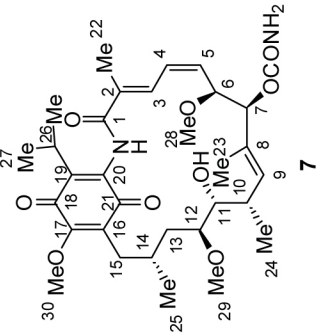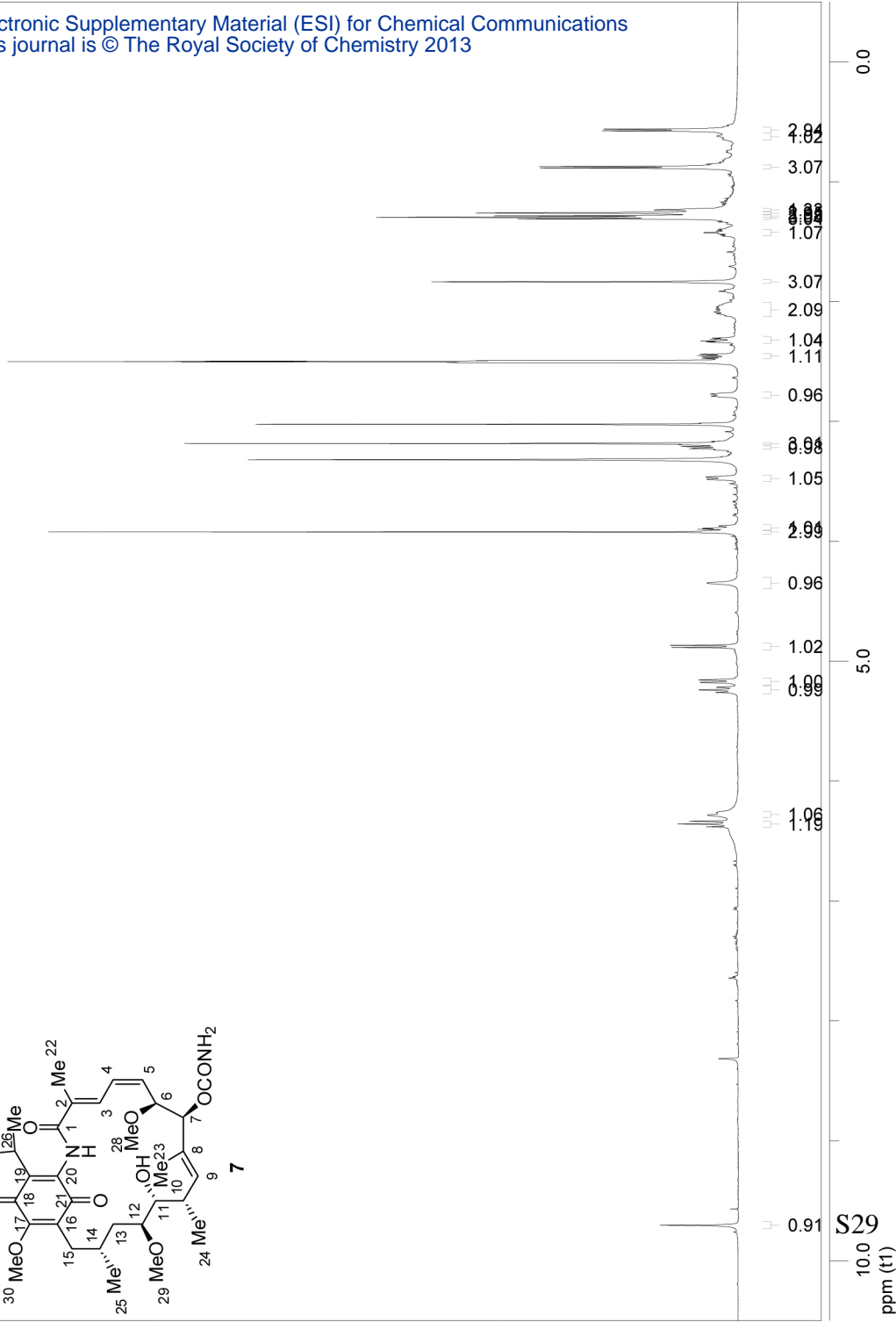

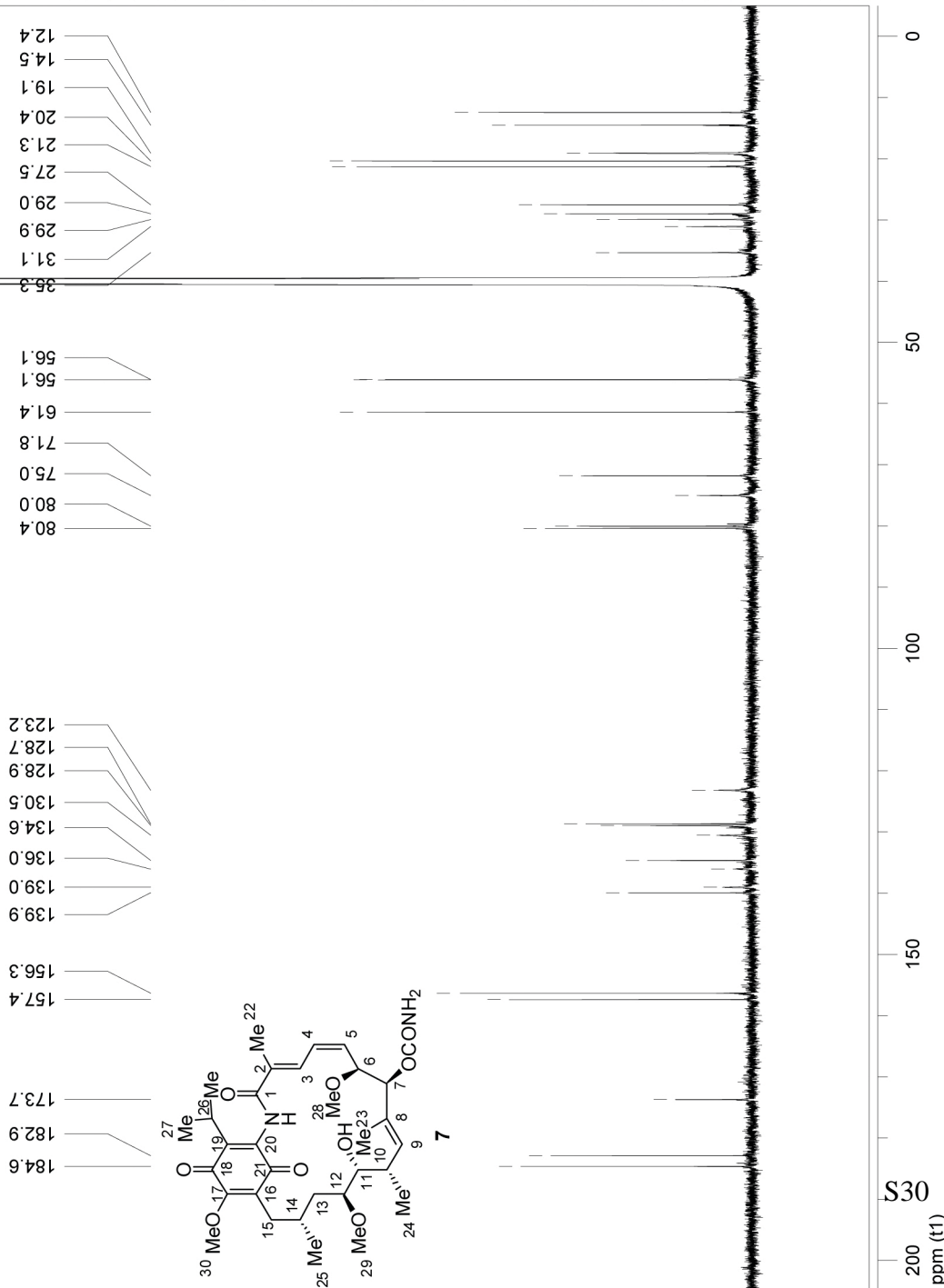

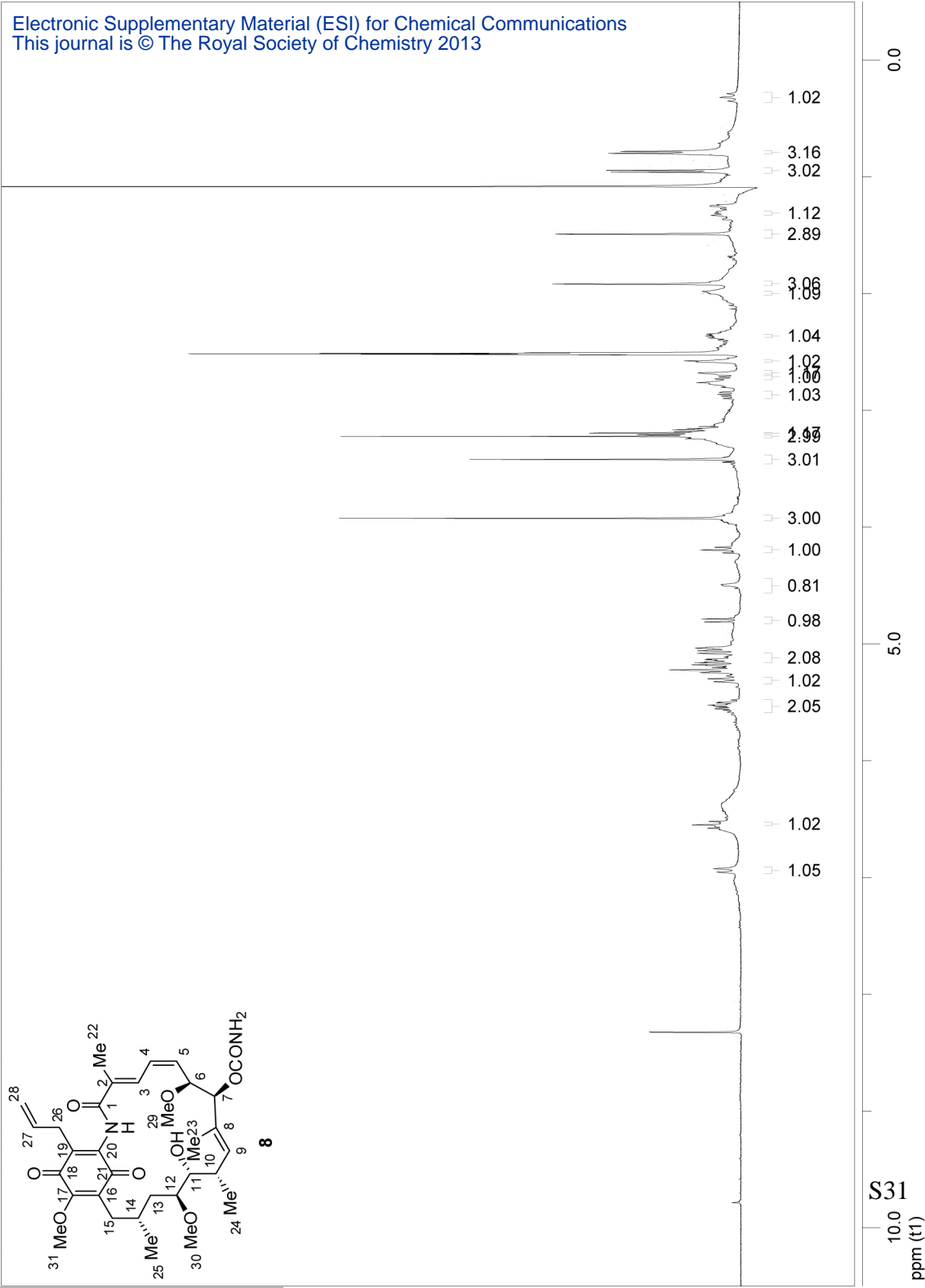

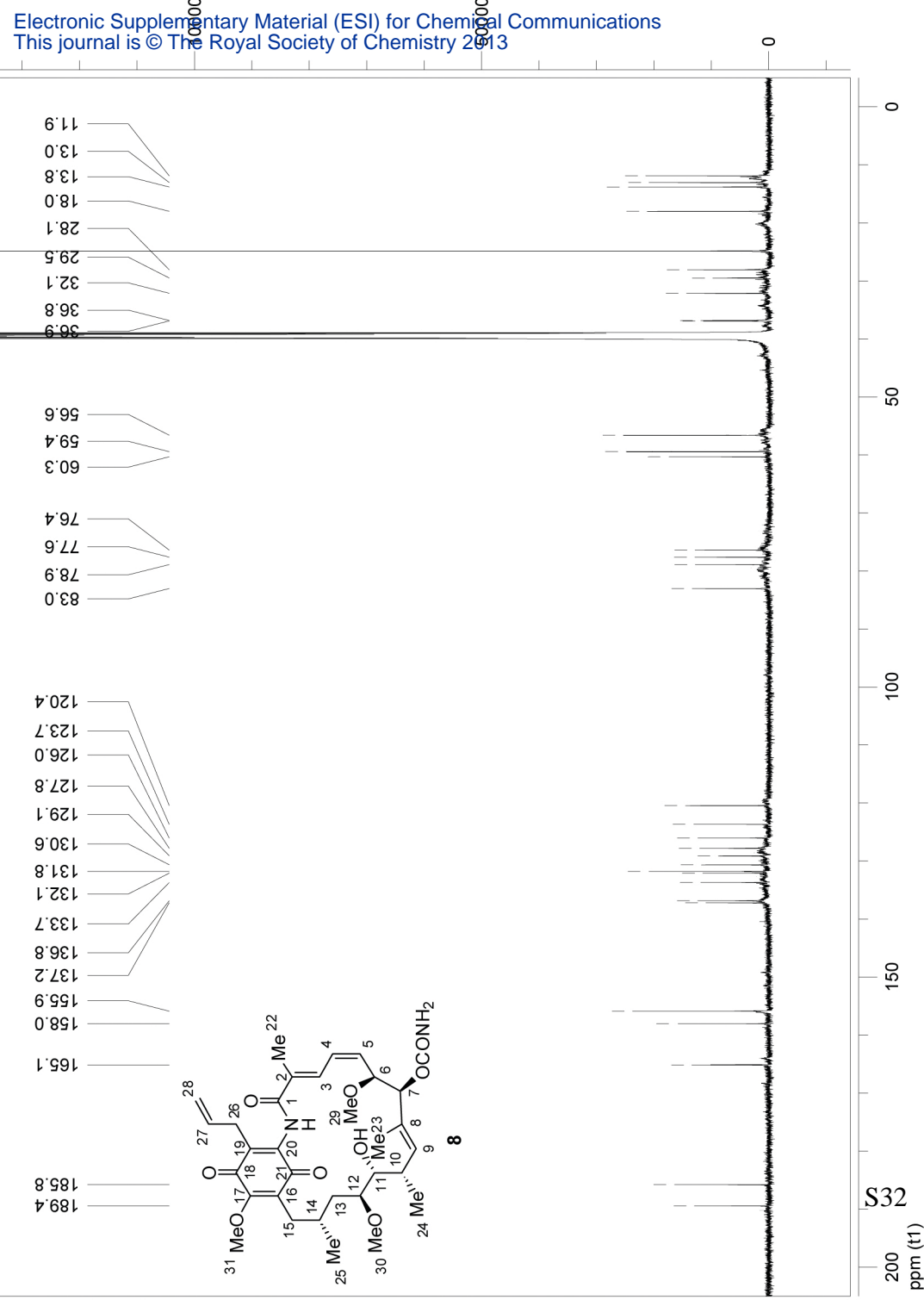

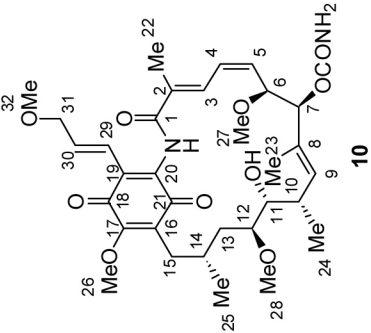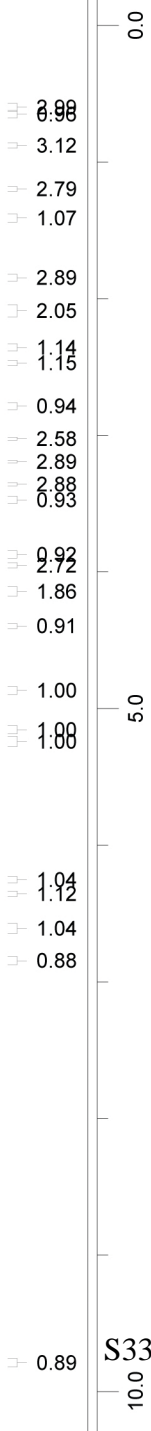

S33  
10.0  
ppm (τ)

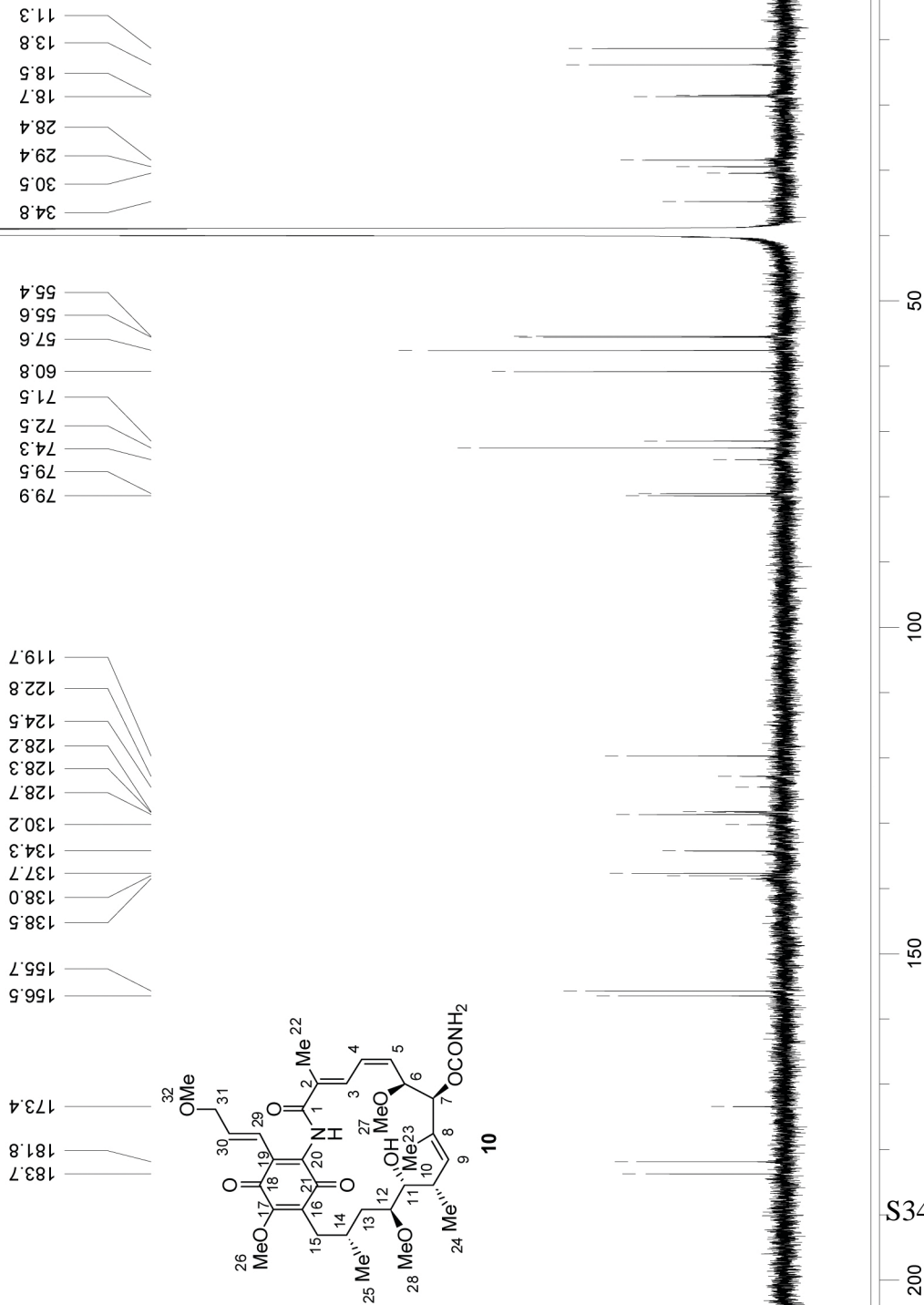

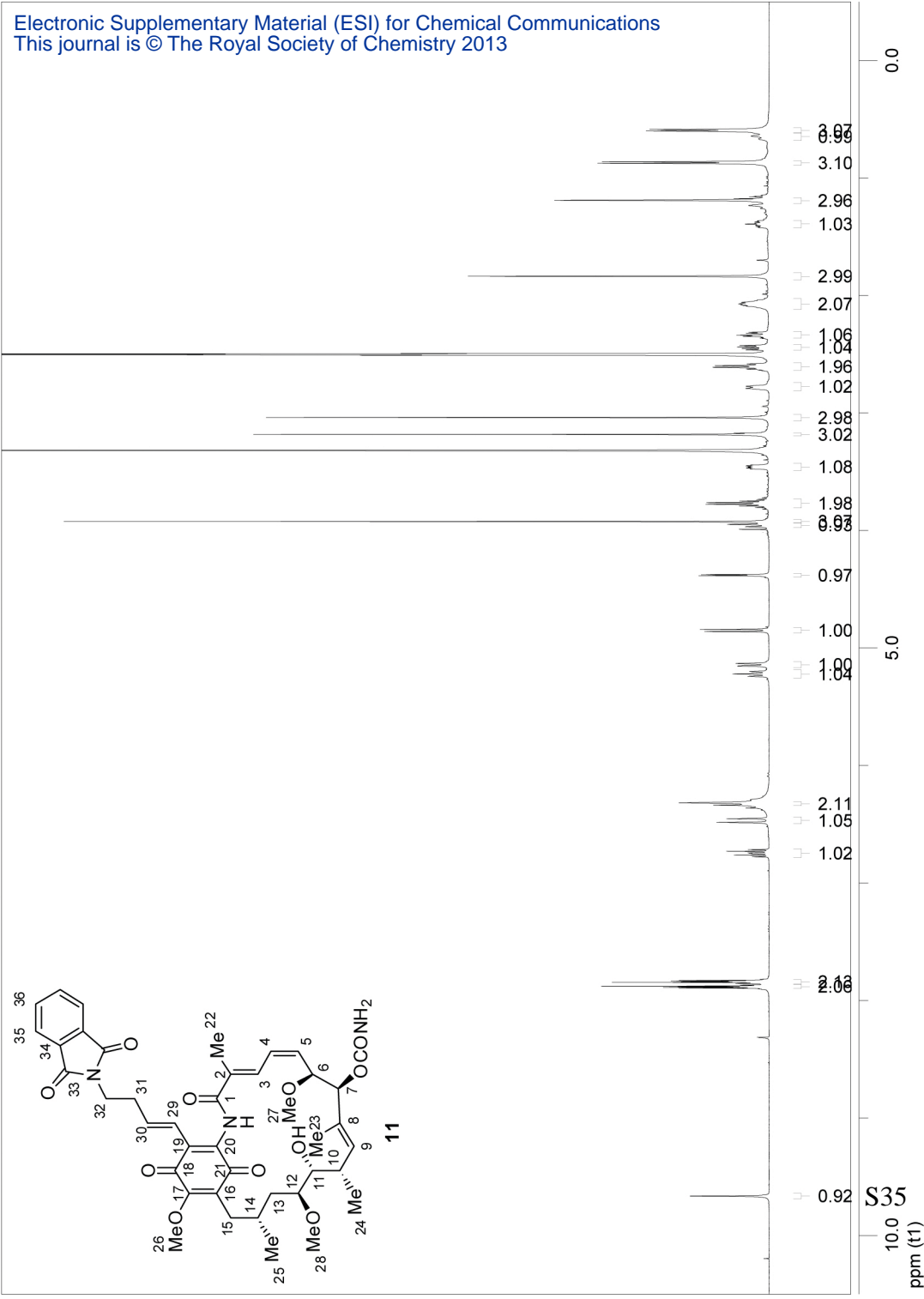

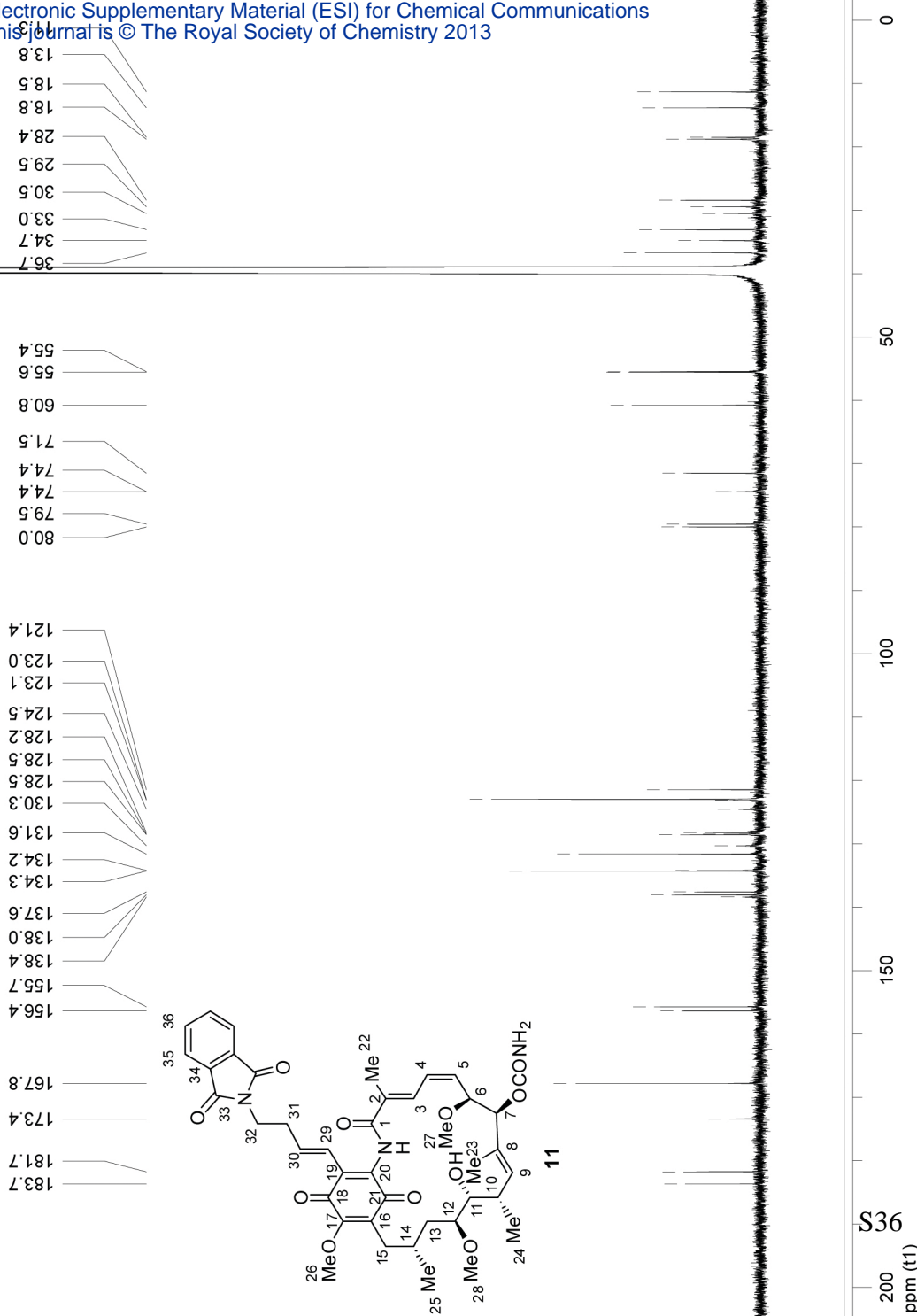

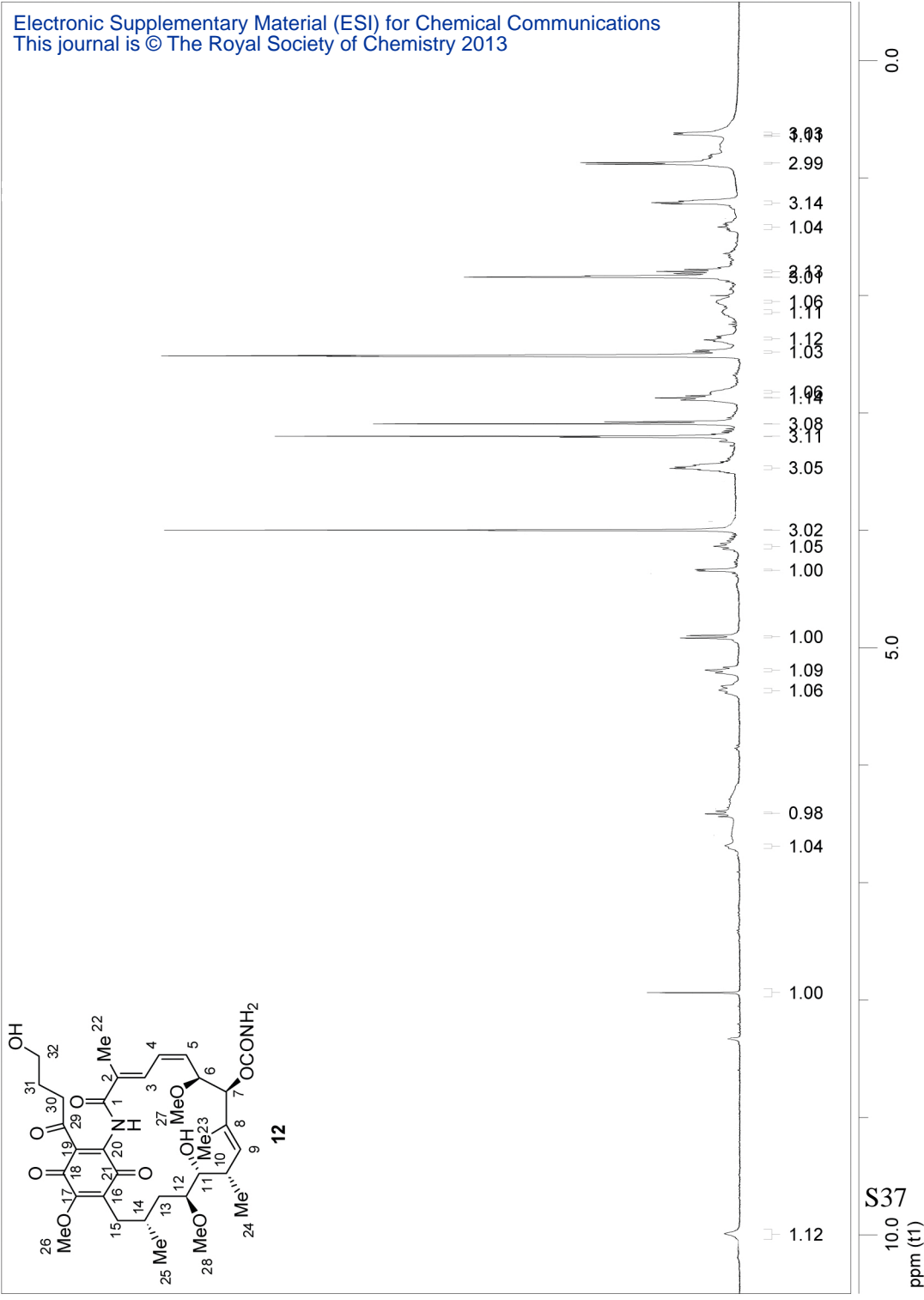

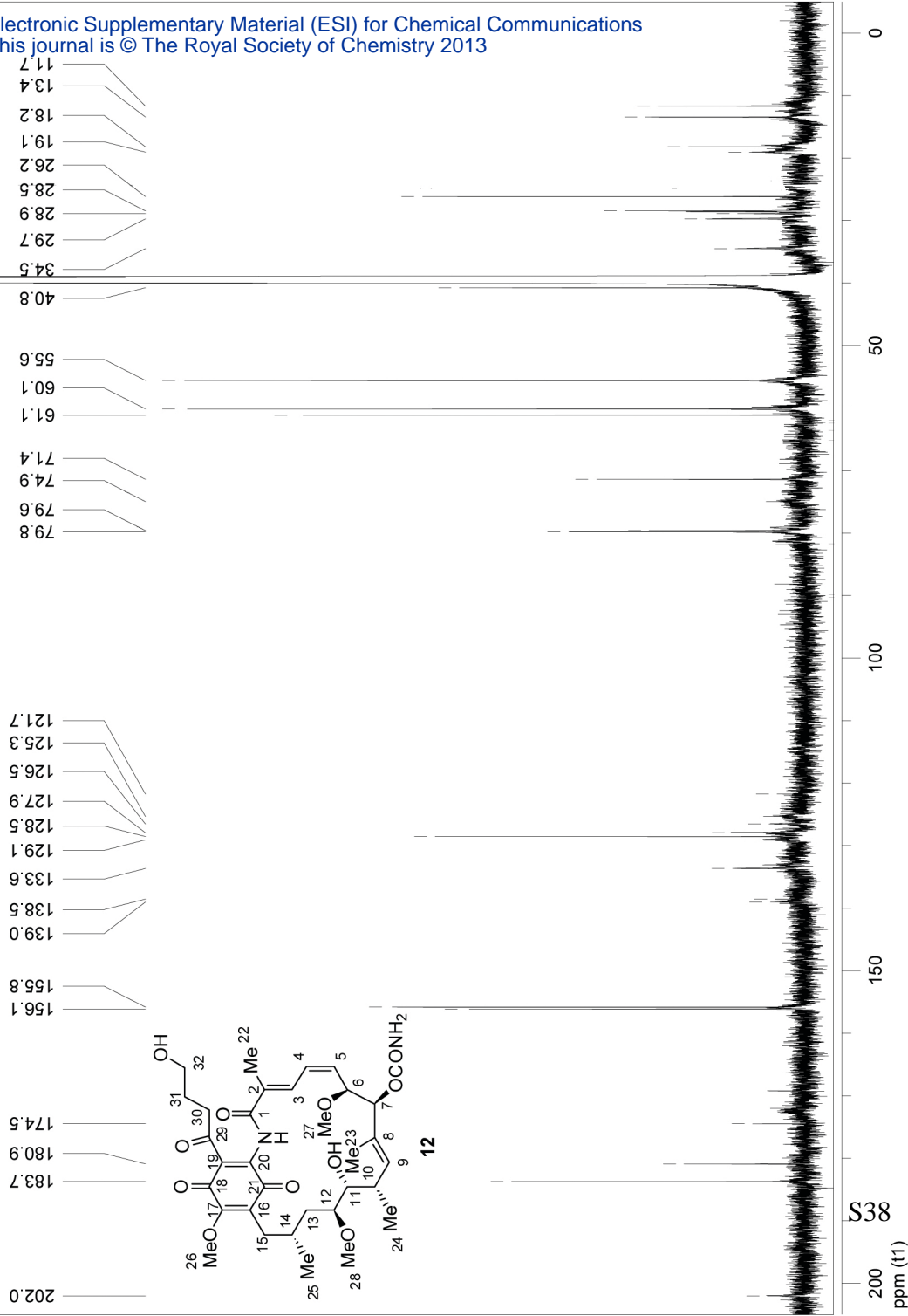

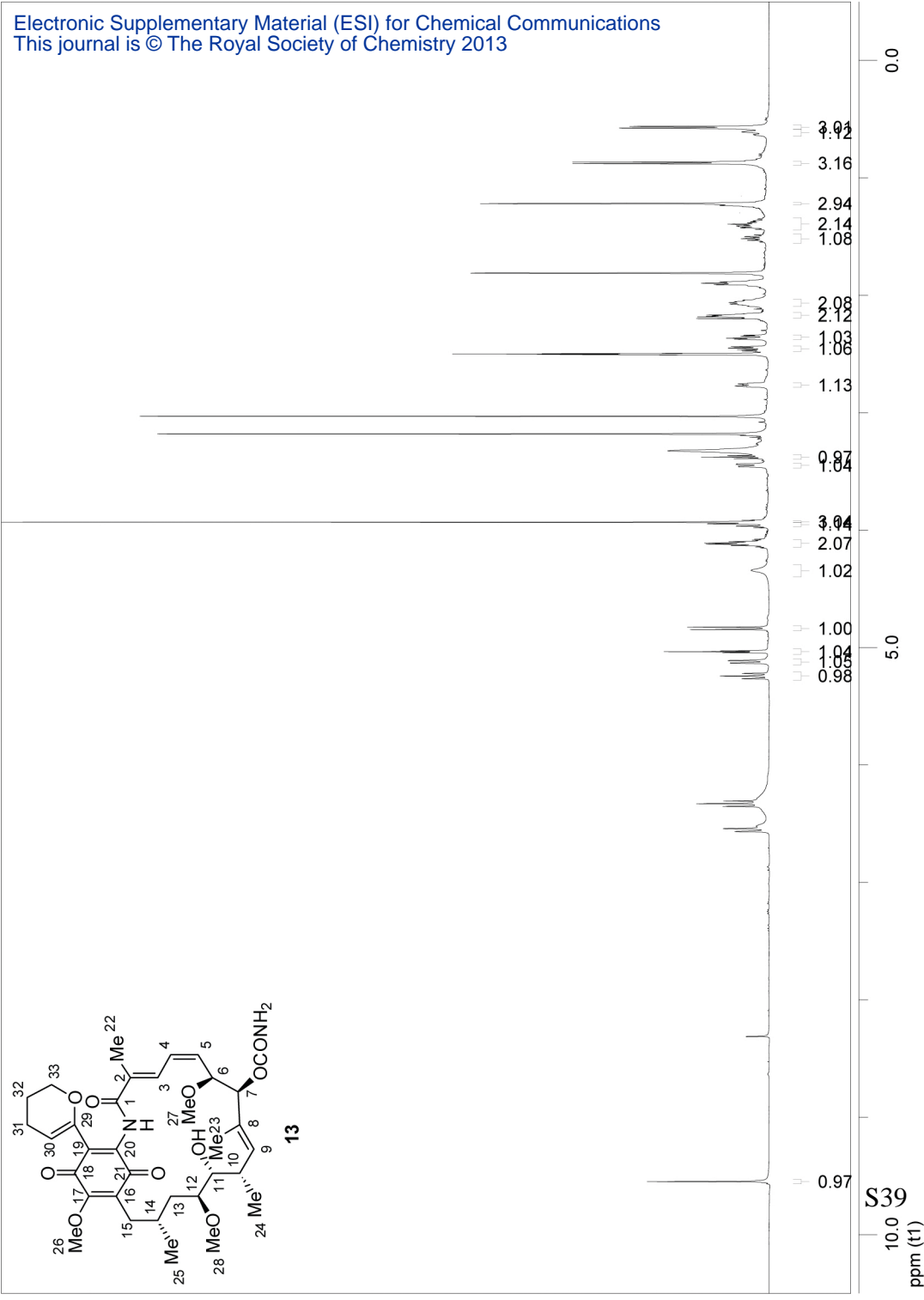

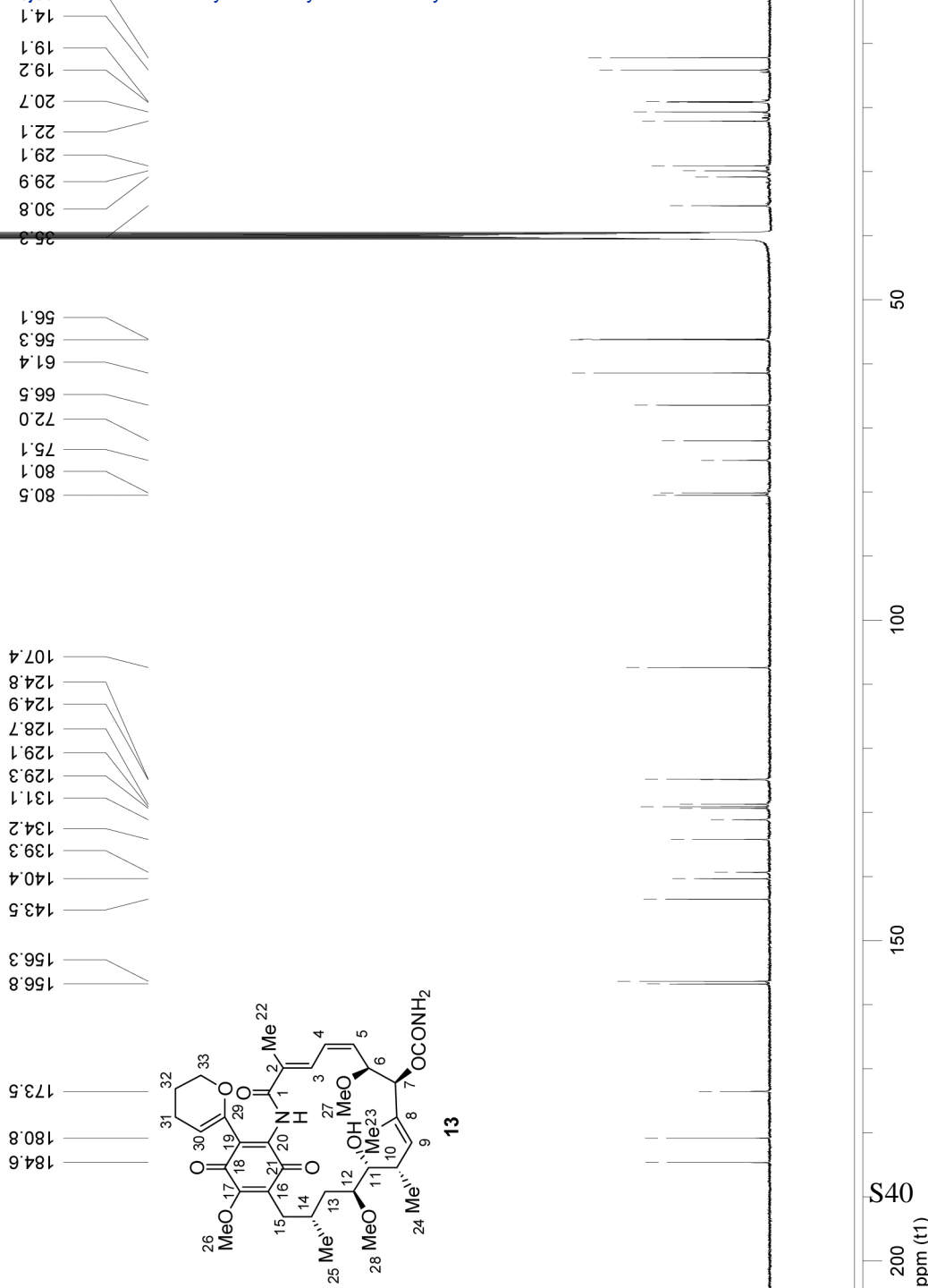

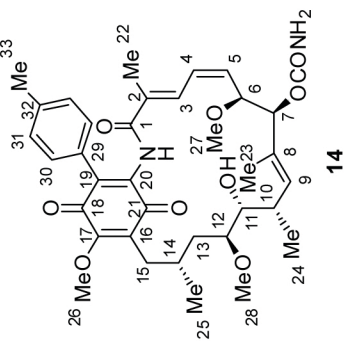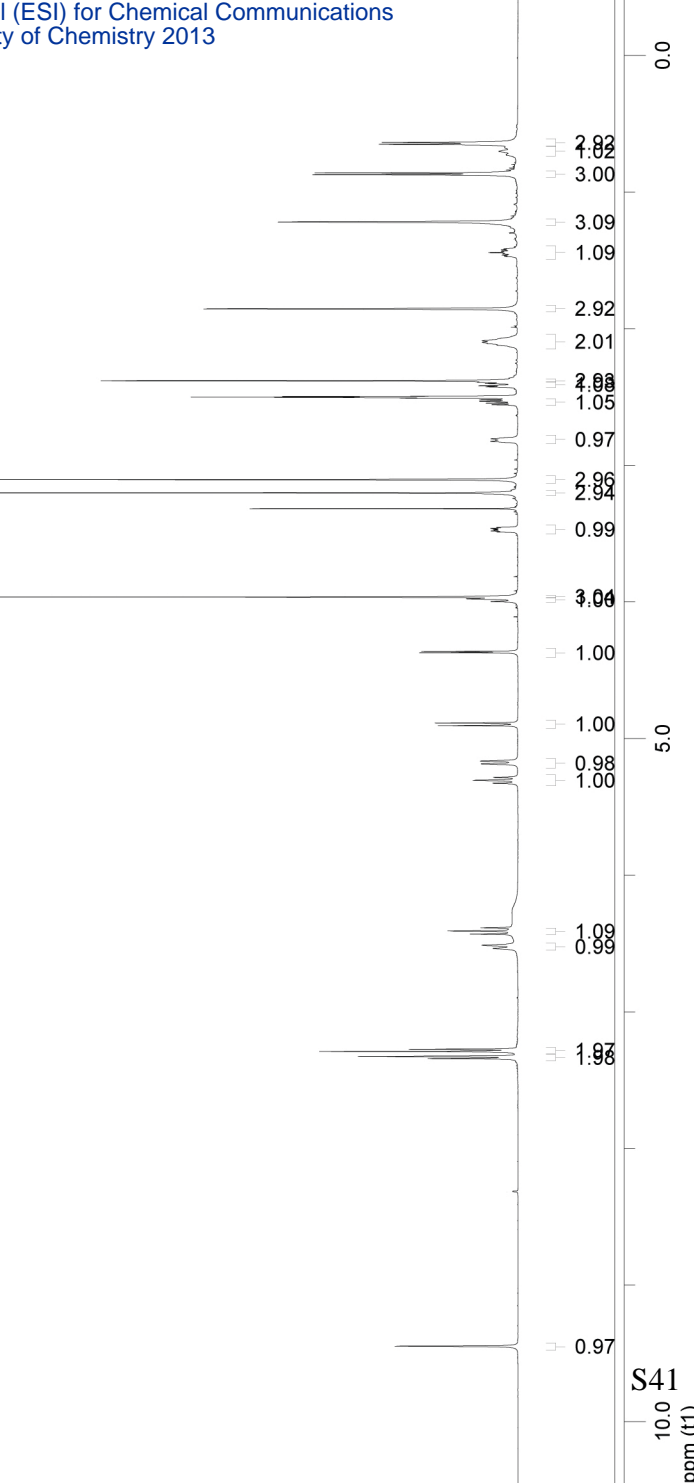

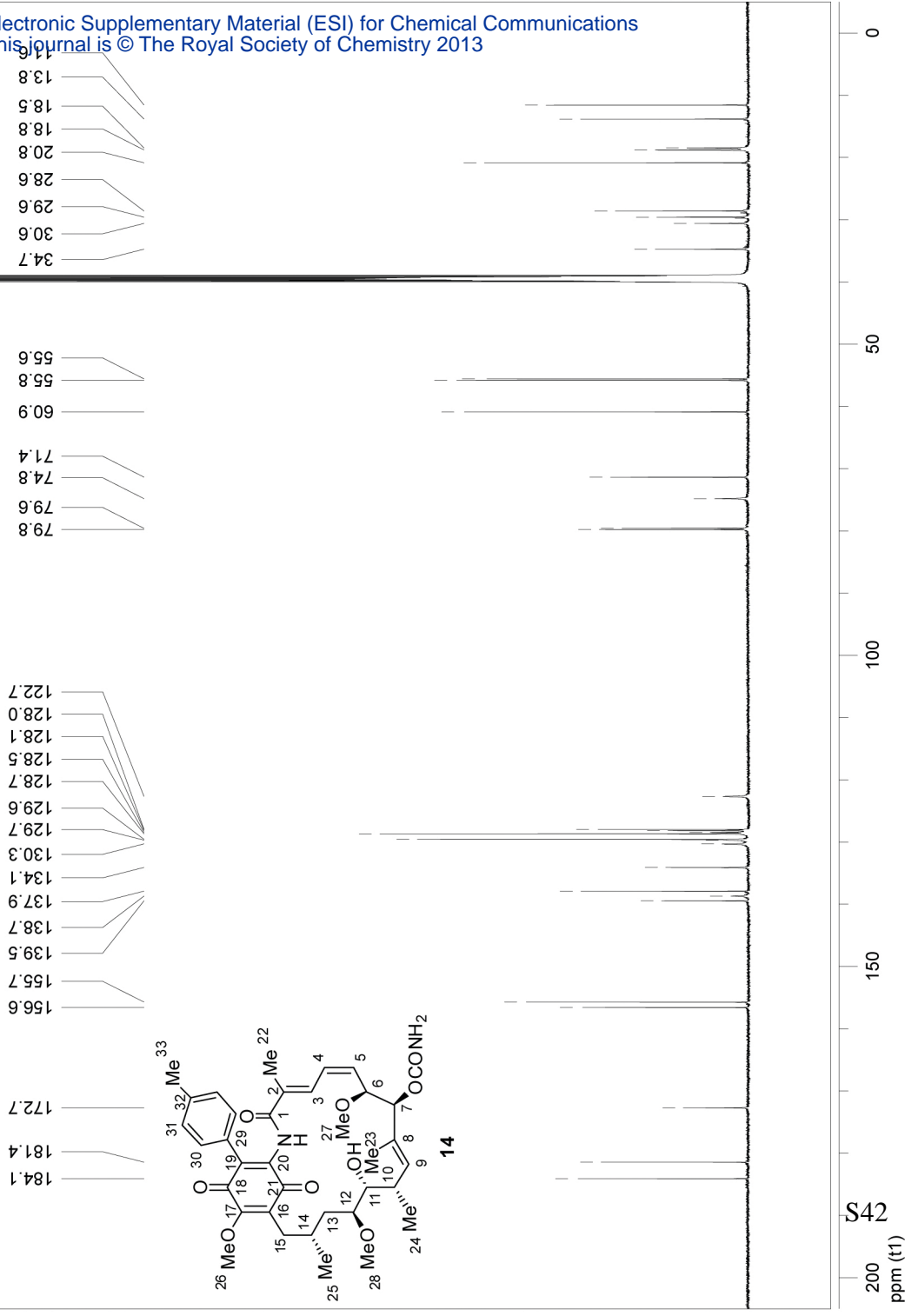

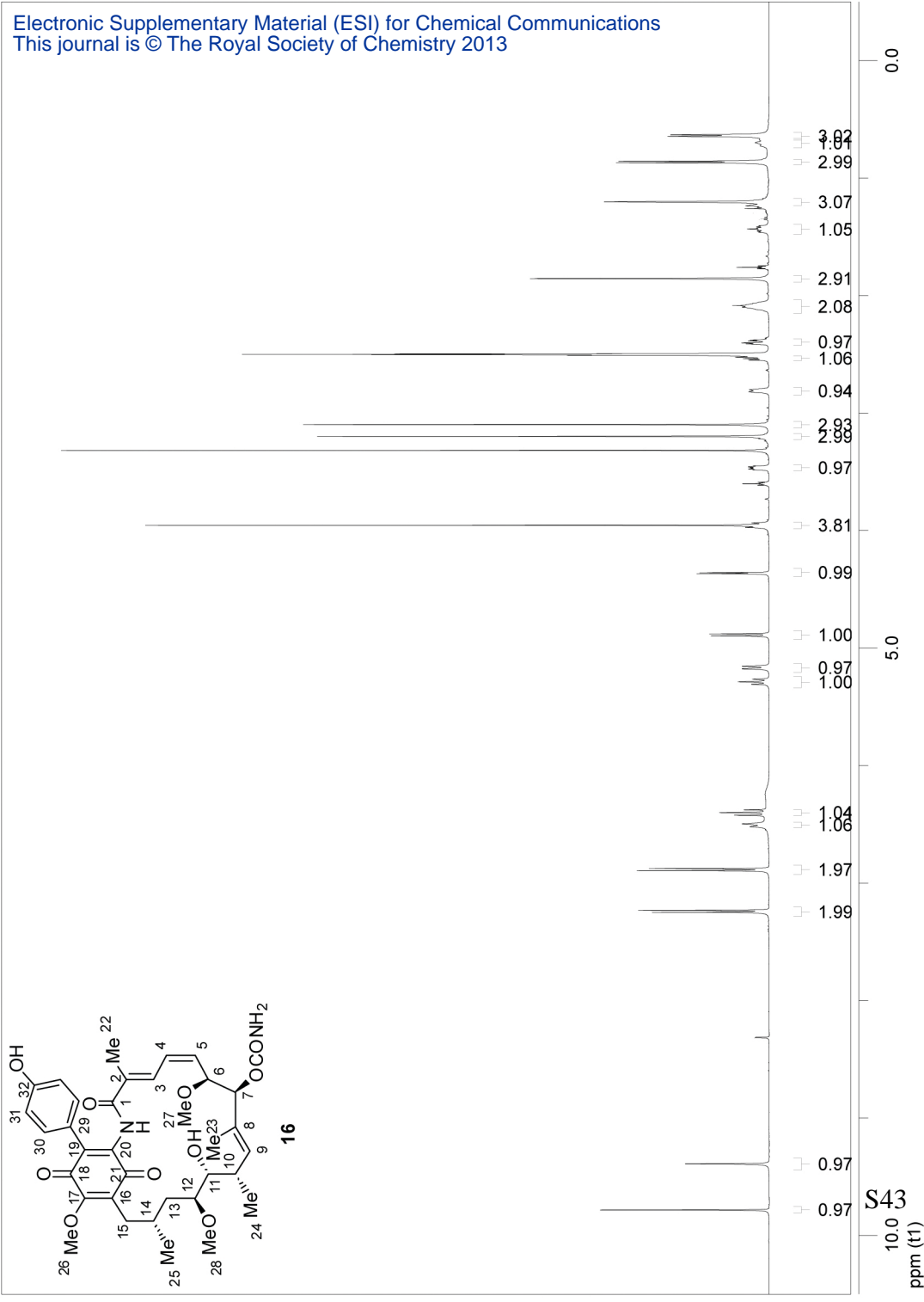

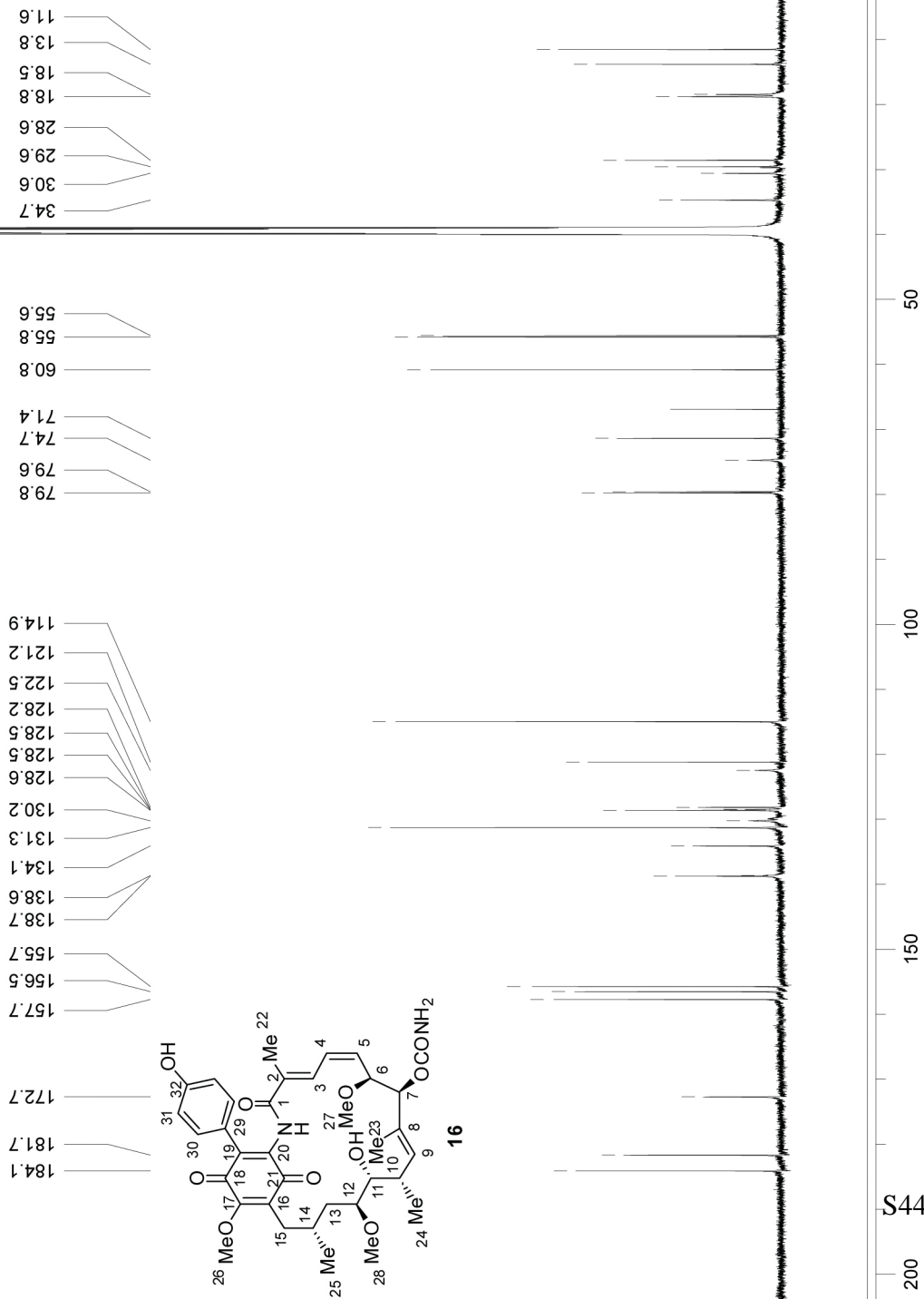

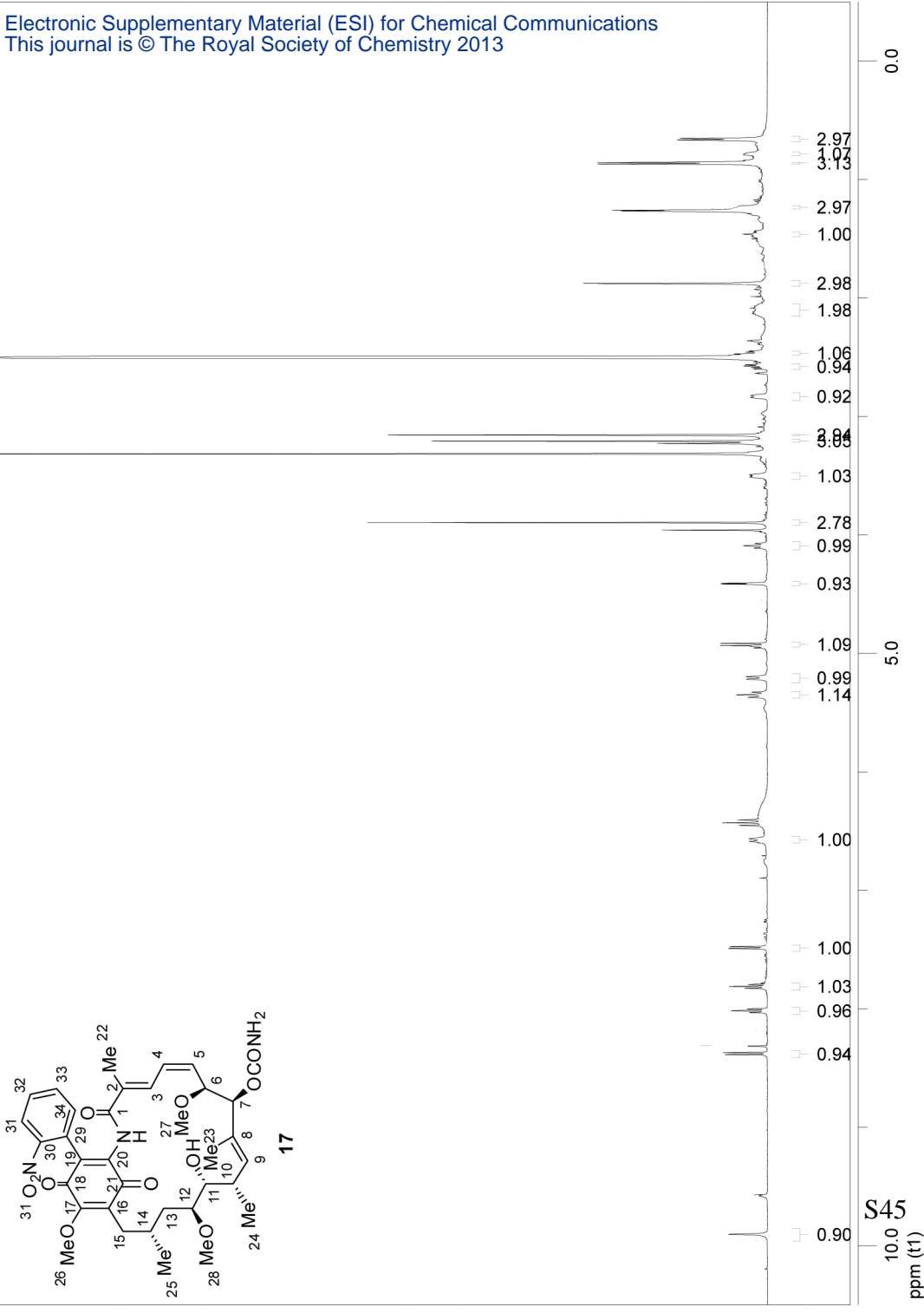

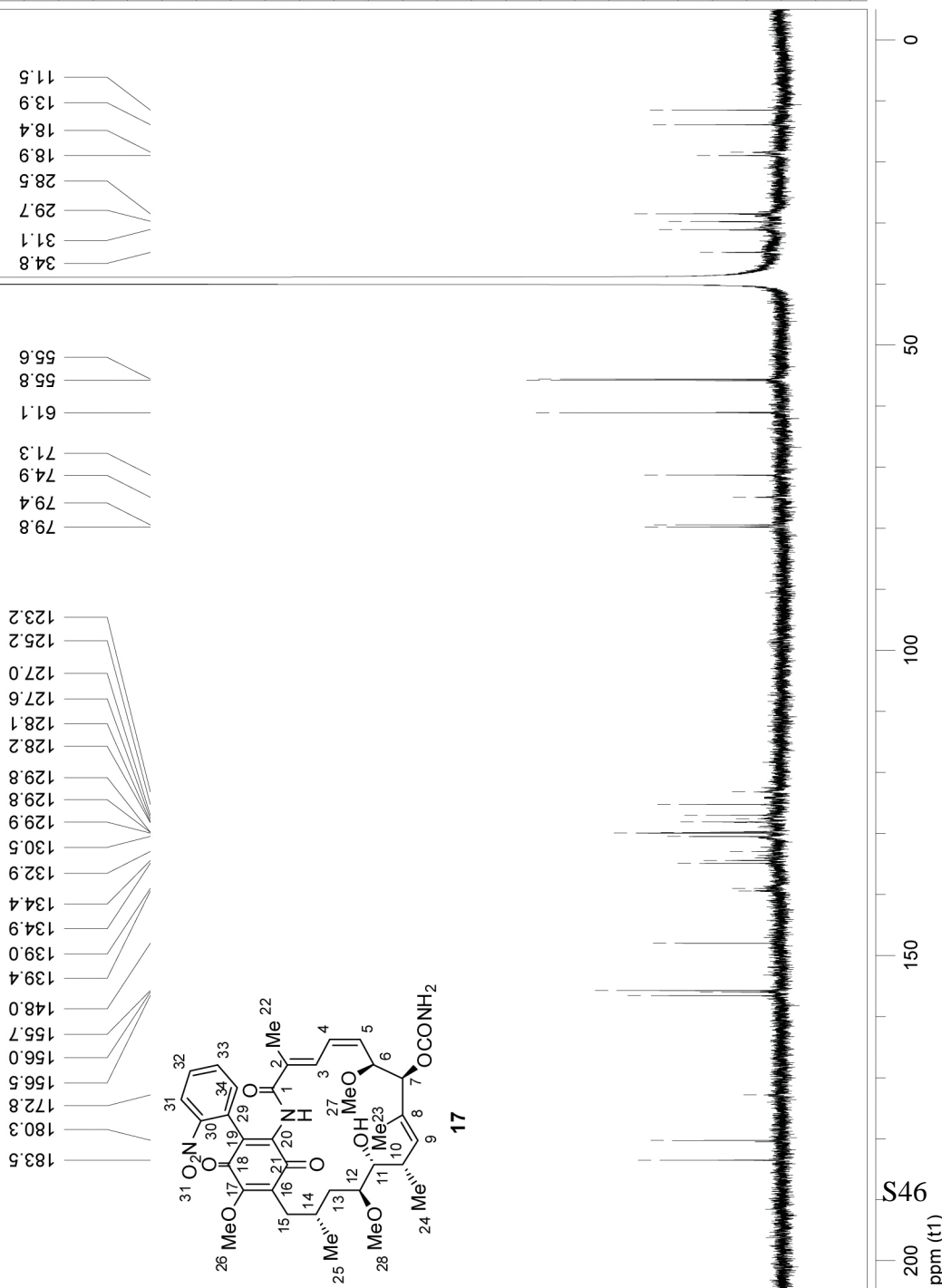

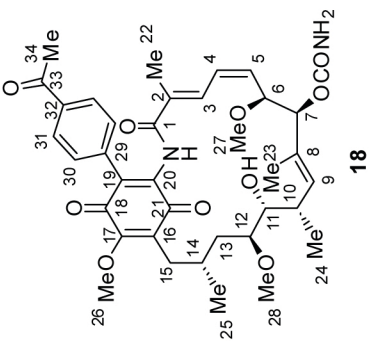

**18**

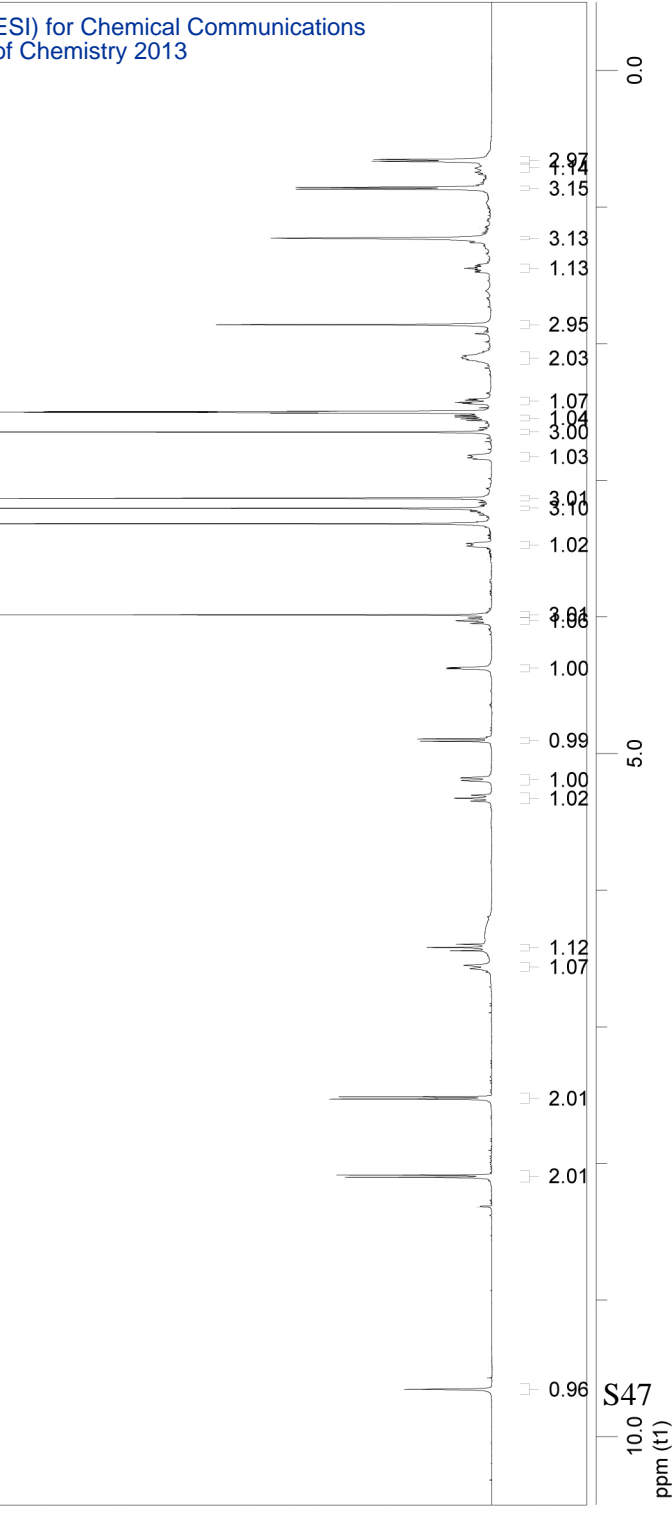

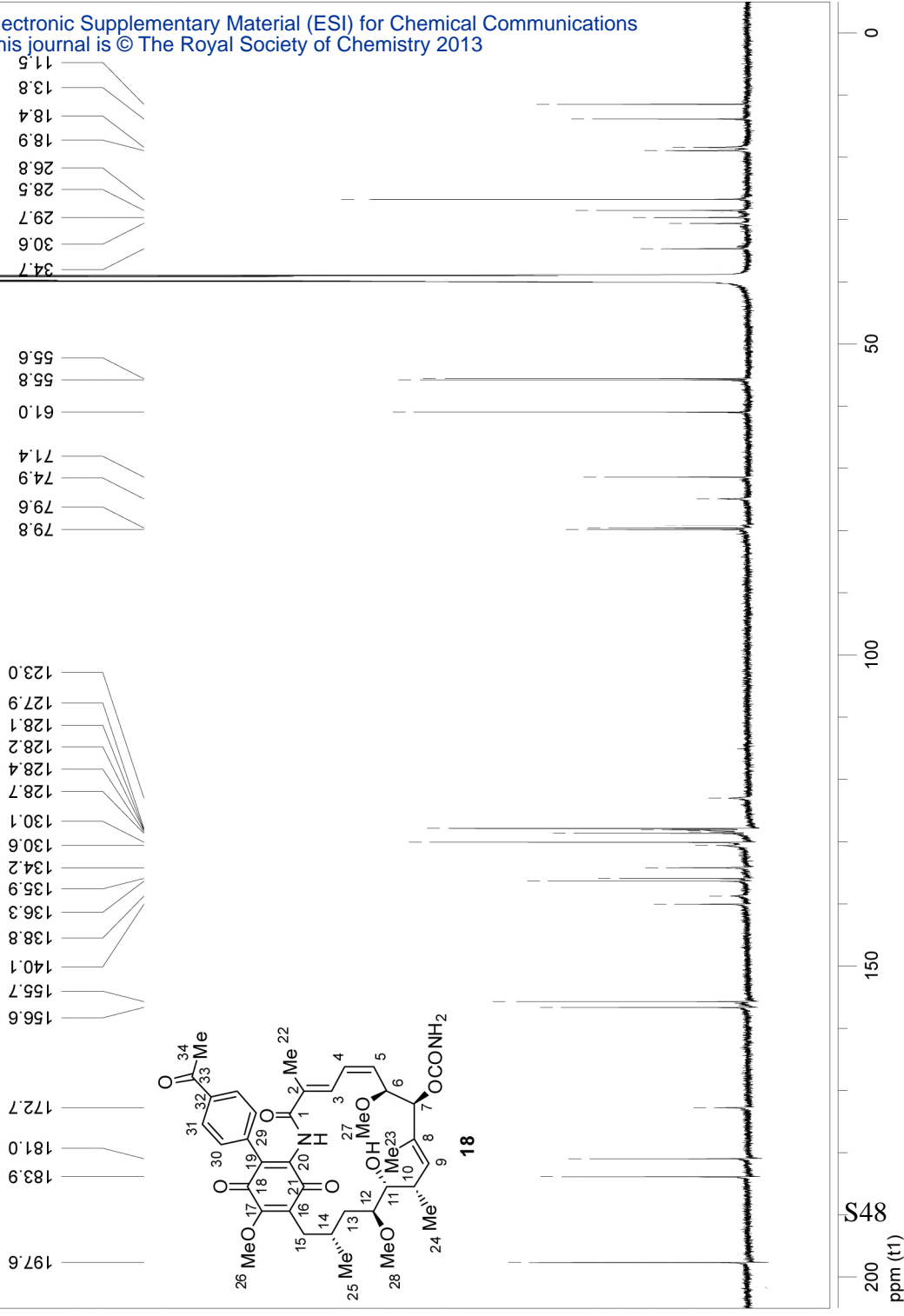

Supplement: Supplementary file 2 [file cc-049-c3cc43457e-s002.pdf]
